# GWAS analysis of handgrip and lower body strength in older adults in the CHARGE consortium

**DOI:** 10.1111/acel.12468

**Published:** 2016-06-21

**Authors:** Amy M. Matteini, Toshiko Tanaka, David Karasik, Gil Atzmon, Wen‐Chi Chou, John D. Eicher, Andrew D. Johnson, Alice M. Arnold, Michele L. Callisaya, Gail Davies, Daniel S. Evans, Birte Holtfreter, Kurt Lohman, Kathryn L. Lunetta, Massimo Mangino, Albert V. Smith, Jennifer A. Smith, Alexander Teumer, Lei Yu, Dan E. Arking, Aron S. Buchman, Lori B. Chibinik, Philip L. De Jager, Denis A. Evans, Jessica D. Faul, Melissa E. Garcia, Irina Gillham‐Nasenya, Vilmundur Gudnason, Albert Hofman, Yi‐Hsiang Hsu, Till Ittermann, Lies Lahousse, David C. Liewald, Yongmei Liu, Lorna Lopez, Fernando Rivadeneira, Jerome I. Rotter, Kristin Siggeirsdottir, John M. Starr, Russell Thomson, Gregory J. Tranah, André G. Uitterlinden, Uwe Völker, Henry Völzke, David R. Weir, Kristine Yaffe, Wei Zhao, Wei Vivian Zhuang, Joseph M. Zmuda, David A. Bennett, Steven R. Cummings, Ian J. Deary, Luigi Ferrucci, Tamara B. Harris, Sharon L. R. Kardia, Thomas Kocher, Stephen B. Kritchevsky, Bruce M. Psaty, Sudha Seshadri, Timothy D. Spector, Velandai K. Srikanth, B. Gwen Windham, M. Carola Zillikens, Anne B. Newman, Jeremy D. Walston, Douglas P. Kiel, Joanne M. Murabito

**Affiliations:** ^1^ Division of Geriatric Medicine and GerontologyJohns Hopkins University School of MedicineBaltimoreMDUSA; ^2^ Longitudinal Studies SectionTranslational Gerontology BranchGerontology Research CenterNational Institute on AgingBaltimoreMDUSA; ^3^ Institute for Aging ResearchHebrew SeniorLifeDepartment of MedicineBeth Israel Deaconess Medical Center and Harvard Medical SchoolBostonMAUSA; ^4^ Faculty of Medicine in the GalileeBar‐Ilan UniversitySafed13010Israel; ^5^ Institute for Aging Research Departments of Medicine and GeneticsAlbert Einstein College of Medicine1300 Morris Park AvenueBronxNYUSA; ^6^ Department of Human BiologyUniversity of HaifaHaifaIsrael; ^7^ National Heart, Lung and Blood InstitutePopulation Sciences BranchBethesdaMDUSA; ^8^ National Heart, Lung and Blood Institute's The Framingham Heart StudyFraminghamMAUSA; ^9^ Department of BiostatisticsUniversity of WashingtonSeattleWAUSA; ^10^ Stroke and Ageing Research GroupDepartment of MedicineSchool of Clinical SciencesMonash UniversityClaytonVic.Australia; ^11^ Menzies Institute for Medical ResearchUniversity of TasmaniaHobartTas.Australia; ^12^ Centre for Cognitive Ageing and Cognitive EpidemiologyUniversity of EdinburghEdinburghUK; ^13^ Department of PsychologyUniversity of EdinburghEdinburghUK; ^14^ California Pacific Medical Center Research InstituteSan FranciscoCAUSA; ^15^ Unit of PeriodontologyDepartment of Restorative Dentistry, Periodontology and EndodontologyCentre of Oral HealthUniversity Medicine GreifswaldGreifswaldGermany; ^16^ Center for Human GeneticsDivision of Public Health SciencesWake Forest School of MedicineWinston‐SalemNCUSA; ^17^ Department of BiostatisticsBoston University School of Public HealthBostonMAUSA; ^18^ Department of Twin Research and Genetic EpidemiologyKing's College LondonLondonUK; ^19^ NIHR Biomedical Research Centre at Guy's and St. Thomas’ Foundation TrustLondonUK; ^20^ Icelandic Heart AssociationKopavogurIceland; ^21^ Department of EpidemiologyUniversity of MichiganAnn ArborMIUSA; ^22^ Institute for Community MedicineUniversity Medicine GreifswaldGreifswaldGermany; ^23^ Rush Alzheimer's Disease CenterRush University Medical CenterChicagoILUSA; ^24^ McKusick‐Nathans Institute of Genetic MedicineJohns Hopkins University School of MedicineBaltimoreMDUSA; ^25^ Department of Neurological SciencesRush University Medical CenterChicagoILUSA; ^26^ Program in Translational NeuroPsychiatric GenomicsDepartment of NeurologyBrigham and Women's HospitalBostonMAUSA; ^27^ Program in Medical and Population GeneticsBroad InstituteCambridgeMAUSA; ^28^ Institute of Healthy Aging and Department of Internal MedicineRush University Medical CenterChicagoILUSA; ^29^ Survey Research CenterInstitute for Social ResearchUniversity of MichiganAnn ArborMIUSA; ^30^ Laboratory of Epidemiology and Population ScienceNational Institute on AgingBethesdaMDUSA; ^31^ University of IcelandReykjavikIceland; ^32^ Department of EpidemiologyErasmus Medical CenterRotterdamthe Netherlands; ^33^ Department of Medicine, Molecular and Integrative Physiological SciencesHarvard School of Public HealthBostonMAUSA; ^34^ Department of Respiratory MedicineGhent University and Ghent University HospitalGhentBelgium; ^35^ Department of Internal MedicineErasmus Medical CenterRotterdamthe Netherlands; ^36^ Netherlands Genomics Initiative (NGI)‐sponsored Netherlands Consortium for Healthy Aging (NCHA)Rotterdamthe Netherlands; ^37^ Division of Genomic Outcome, Departments of Pediatrics and MedicineInstitute for Translational Genomics and Population SciencesLos Angeles Biomedical Research Institute at Harbor‐UCLA Medical CenterUniversity of California Los AngelesLos AngelesCAUSA; ^38^ Alzheimer Scotland Dementia Research CentreUniversity of EdinburghEdinburghUK; ^39^ Interfaculty Institute for Genetics and Functional GenomicsUniversity Medicine GreifswaldGreifswaldGermany; ^40^ German Center for Cardiovascular Research (DZHK)GreifswaldGermany; ^41^ German Center for Diabetes Research (DZD)GreifswaldGermany; ^42^ Departments of Neurology, Psychiatry and Epidemiology & BiostatisticsUniversity of California, San Francisco and the San Francisco Veterans Affairs Medical CenterSan FranciscoCAUSA; ^43^ Public Health ProgramCenter for Health Policy and EthicsCreighton University School of MedicineOmahaNEUSA; ^44^ Department of EpidemiologyUniversity of PittsburghPittsburghPAUSA; ^45^ Department of PsychologyUniversity of EdinburghEdinburghUK; ^46^ Laboratory of Epidemiology and Population ScienceNIABethesdaMDUSA; ^47^ Sticht Center on AgingWake Forest School of MedicineWinston‐SalemNCUSA; ^48^ Cardiovascular Health Research Unit and Department of MedicineUniversity of Washington and Group Health Research InstituteGroup Health CooperativeSeattleWAUSA; ^49^ Department of NeurologyBoston University School of MedicineBostonMAUSA; ^50^ Department of Medicine/Division of GeriatricsUniversity of Mississippi Medical CenterJacksonMSUSA; ^51^ Department of MedicineBoston University School of MedicineBostonMAUSA

**Keywords:** aging, genomewide association, meta‐analysis, muscle strength, older adults, SNP

## Abstract

Decline in muscle strength with aging is an important predictor of health trajectory in the elderly. Several factors, including genetics, are proposed contributors to variability in muscle strength. To identify genetic contributors to muscle strength, a meta‐analysis of genomewide association studies of handgrip was conducted. Grip strength was measured using a handheld dynamometer in 27 581 individuals of European descent over 65 years of age from 14 cohort studies. Genomewide association analysis was conducted on ~2.7 million imputed and genotyped variants (SNPs). Replication of the most significant findings was conducted using data from 6393 individuals from three cohorts. GWAS of lower body strength was also characterized in a subset of cohorts. Two genomewide significant (*P*‐value< 5 × 10^−8^) and 39 suggestive (*P*‐value< 5 × 10^−5^) associations were observed from meta‐analysis of the discovery cohorts. After meta‐analysis with replication cohorts, genomewide significant association was observed for rs752045 on chromosome 8 (β = 0.47, SE = 0.08, *P*‐value = 5.20 × 10^−10^). This SNP is mapped to an intergenic region and is located within an accessible chromatin region (DNase hypersensitivity site) in skeletal muscle myotubes differentiated from the human skeletal muscle myoblasts cell line. This locus alters a binding motif of the CCAAT/enhancer‐binding protein‐β (CEBPB) that is implicated in muscle repair mechanisms. GWAS of lower body strength did not yield significant results. A common genetic variant in a chromosomal region that regulates myotube differentiation and muscle repair may contribute to variability in grip strength in the elderly. Further studies are needed to uncover the mechanisms that link this genetic variant with muscle strength.

## Introduction

Loss of muscle strength, ‘dynapenia’, is a common characteristic of aging and is associated with increased risk of frailty, falls, hospitalizations and mortality (Moreland *et al*., 2004; Xue *et al*., 2010; Marsh *et al*., 2011). In particular, handgrip strength is found to be predictive of overall and exceptional survival (Willcox *et al*., 2006) and other key age‐related outcomes (Marsh *et al*., 2011; McLean *et al*., 2014). For example, poor handgrip strength among healthy middle‐aged subjects was found to significantly predict functional limitations and disability 25 years later (Rantanen *et al*., 1999). The biology that drives muscle strength decline is complex, with hormonal changes, inflammatory pathway activation, mitochondrial physiology, malnutrition, and exercise all likely playing a role (Walston, 2012; Gonzalez‐Freire *et al*., 2014). Further identification of biologically relevant pathways that influence muscle strength maintenance and decline could be important in the development of future treatment or prevention strategies. Hence, genetic approaches to the identification of novel biology may be helpful.

The heritability of muscle strength in older adults has been estimated to be between 40 and 65% (Tiainen *et al*., 2004; Matteini *et al*., 2010). Previously published reports have been limited to candidate gene analyses in small cohorts of older adults (Arking *et al*., 2006; Serena Dato *et al*., 2012; S Dato *et al*., 2014). These studies have highlighted potentially important biologic pathways associated with handgrip strength but have been unable to identify a significant replicated locus. In spite of the importance of this phenotype for health and function, to date, no genomewide association study (GWAS) has been published on handgrip strength.

Because of the large, well‐characterized cohorts represented in the CHARGE consortium, grip strength and genomewide genotype data from 17 cohort studies (14 discovery and three replication cohorts) of older adults were included in this meta‐analysis. We sought to identify potential genetic influences that underlie measures of strength in adults aged 65 years and older.

## Results

### Discovery set

**Figure 1:**
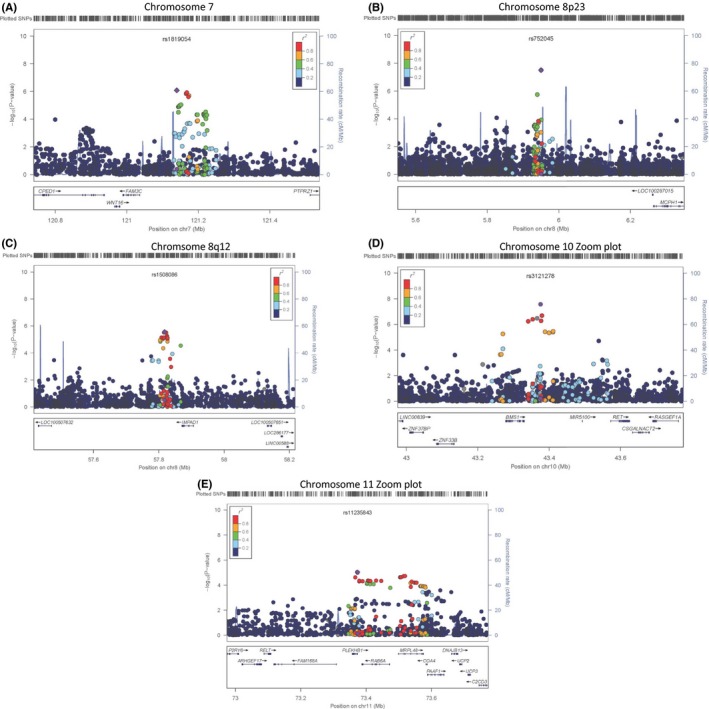
Regional association plots for the most significant associations from the meta‐analysis of handgrip strength in the discovery set. The figures display –log10 *P*‐values for SNPs that passed quality control for the analysis of handgrip strength for locus on (A) chromosome 7, (B) chromosome 8p23, (C) chromosome 8q12, (D) chromosome 10, and (E) chromosome 11. The degree of linkage disequilibrium (*r* ^2^) is displayed in the following categories: *r* ^2^ ≥ 0.8, ≥ 0.6, ≥0.4, ≥0.2, and ≥0.

A genomewide meta‐analysis included 27 581 community‐dwelling men and women of European ancestry from a discovery set of 14 participating cohorts. On average across the cohorts, there were 2 725 778 SNPs analyzed, with SNPs analyzed per cohort ranging from 2 332 998 to 4 930 728. Sample size and cohort characteristics are found in Table S1 (Supporting information). There were no significant differences in age, strength, or gender distributions between the discovery and replication cohorts. Q‐Q and Manhattan plots are shown in Figs S1, S2 (Supporting information). In the discovery set meta‐analysis, 2 SNPs reached genomewide significance (rs3121278 chr10: *P*‐value = 2.68 × 10^−8^ and rs752045 chr8: *P*‐value = 3.09 × 10^−8^). An additional 39 SNPs reached suggestive significance in eight regions on chromosomes 1 (one SNP), 5 (two highly correlated SNPs), seven (seven SNPs), 8p23 (two SNPs), 8q12 (14 SNPs), 10 (11 SNPs), 11 (three SNPs), and 12 (one SNP) (Table S4). Chromosomes 1, 5, and 12 loci were not pursued in subsequent analysis due to the fact that there was only a single SNP in the locus with suggestive significance. The five regions that remained suggestive are intergenic. Table 1 shows the lead SNP per region with meta‐analyzed results from discovery, replication as well as combined discovery and replication cohorts. Regional plots (created using Locus zoom http://csg.sph.umich.edu/locuszoom/) are displayed in Fig. 1.

**Table 1:** Top SNP in each region with suggestive association with handgrip in discovery and replication sets

SNP	Chr	Position	Effect /Noneffect Allele	Frequency of Effect Allele	Gene Structure	Most Proximal Gene(s)	Distance to gene (kb)	Discovery Set (*n* = 27581)		Replication Set (*n* = 6363)		Discovery + Replication Set (*n* = 34 910)	
								Beta (SE)	*P*‐value_disc_	Beta (SE)	*P*‐value_rep_	Beta (SE)	*P*‐value _disc+rep_
rs1819054	7	120926996	G/A	0.40	Intergenic	*FAM3C PTPRZ1*	103(37)	0.27 (0.06)	8.23 × 10^−7^	0.15 (0.13)	0.24	0.25 (0.05)	6.13 × 10^−7^
rs752045	8	5937538	G/A	0.18	Intergenic	*CSMD1 LOC100287015*	1098(311)	0.47 (0.09)	3.09 × 10^−8^	0.45 (0.16)	4.80E‐03	0.47 (0.08)	5.20 × 10^−10^
rs1508086	8	57980052	T/C	0.44	Intergenic	*LINC00968 IMPAD1*	345(53)	0.25 (0.05)	2.71 × 10^−6^	0.09 (0.12)	0.45	0.22 (0.05)	4.21 × 10^−6^
rs3121278	10	42695652	T/G	0.18	Intergenic	*BMS1L LINC01264*	45(98)	−0.39 (0.07)	2.68 × 10^−8^	0.38 (0.15)	1.00E‐02	−0.26 (0.06)	6.18 × 10^−5^
rs11235843	11	73051644	A/G	0.10	Downstream	*PLEKHB1*		−0.38 (0.08)	9.23 × 10^−6^	−0.40 (0.20)	4.70E‐02	−0.38 (0.08)	1.19 × 10^−6^

### Replication cohorts

Significant and suggestive SNPs on chromosomes 7, 8p23, 8q12, 10, and 11 were tested in the replication cohorts and in the combined discovery/replication set. First, the most significant discovery SNP, rs3121278, was significant in the replication (*P*‐value_rep _= 0.01), yet the effect was in the opposite direction from the discovery set resulting in a decrease in significance in the combined analysis (*P*‐value_disc+rep _= 6.18 × 10^−5^). Next, SNP rs752045 on chromosome 8p23 showed an association with grip strength upon replication and the direction was consistent with that of the discovery set (*P*‐value_rep_ = 4.80 × 10^−3^), leading to increased significance in the combined set (*P*‐value_disc+rep _= 5.20 × 10^−10^). Likewise, the second best SNP on chromosome 11 rs11235843 showed consistent direction and magnitude of effect in the replication cohorts (*P*‐value_rep_ = 4.70 × 10^−2^) and significance in the combined set increased (*P*‐value_disc+rep _= 1.19 × 10^−6^), although it still failed to reach the preset threshold for genomewide significance. Lastly, SNPs in suggestive areas of chromosome 7 and 8q12 showed no effect upon replication. Combined results from these regions showed slightly decreased significance, although *P*‐values were still in the range of suggestive association.

### Lower body strength

A meta‐analysis of genomewide association analysis of lower body strength was conducted as a secondary muscle strength phenotype. There were no genomewide significant associations identified (Fig. S3). The most significant association was observed for rs16831 on chr11 (*P* = 6.07 × 10^−7^; Table S5). The closest gene was an uncharacterized gene LOC101929497 approximately 187 Mb away. We also looked up the top signals from the grip strength analysis; however, these loci were not significantly associated with lower body strength (*P* > 0.05; Table S6).

### Functional annotation

Results from the functional annotation analysis are shown in Table 2. SNPs in the chromosome 7, 10, and 11 regions showed direct links to the regulatory chromatin states in muscle tissue or accessible chromatin states according to ChIP‐seq and DNase‐seq data. First, top discovery SNPs rs3121278 and rs752045 were located within accessible chromatin regions in skeletal muscle myotubes differentiated from the skeletal muscle myoblast (HSMM) cell lines. The suggestive SNP rs2796549 also was located within an accessible chromatin region in skeletal muscle myoblasts. Next, the three suggestive chromosome 11 SNPs localized to motifs predicted to be regulatory elements, promoters, and enhancers, in skeletal muscle myoblasts. The top suggestive chromosome 7 SNP rs1819054 was not shown to affect gene regulatory elements in muscle‐related tissues; however, three SNPs within the region were predicted to localize in regulatory enhancers in skeletal muscle myoblasts. This chromosome 7 locus was significantly enriched for enhancer/promoter elements in muscle cells compared with other muscle types (*P*‐value = 9.9 × 10^−5^). Suggestive SNPs on chromosomes 7, 8p12, and 10 were also predicted to alter binding motifs of the CCAAT/enhancer‐binding protein beta, delta, and gamma family (CEBPB, CEBPD, and CEBPG), zinc finger protein 263 (ZNF263), and the nuclear factor kappa beta (NF‐kB).

**Table 2:** Functional annotations of the GWAS SNPs by histone marks, ChIP‐seq, and DNase‐seq from ENCODE project and epigenetic roadmap project

SNP	Chr	Position (hg18)	Gene Structure	Closest Gene (kb away)	Functional annotation results			Enhancer/promoter enrichment in muscle cells^a^	
					Regulatory motifs altered^b^	Muscle‐related DNase‐seq	Muscle‐related regulatory chromatin states	# SNPs in LD^c^	Permutation *P*‐values^d^
rs3857836	7	120931488	Intergenic	FAM3C (108) PTPRZ1 (369)			Weak enhancer in skeletal muscle myoblasts^1^	33	9.9 × 10^−5^
rs11761290	7	120932659	Intergenic	FAM3C (109) PTPRZ1 (368)			Strong enhancer in skeletal muscle myoblasts^1^ and skeletal muscle^2^	33	9.9 × 10^−5^
rs10228676	7	120932913	Intergenic	FAM3C (109) PTPRZ1 (368)	CEBPG; Hoxa5		Weak enhancer in skeletal muscle myoblasts^1^	33	9.9 × 10^−5^
rs1013711	7	120943334	Intergenic	FAM3C (120) PTPRZ1 (357)	CEBPB; CEBPD		Weak enhancer in colon smooth muscle^2^	8	9.9 × 10^−5^
rs1528351	7	120955111	Intergenic	FAM3C (131) PTPRZ1 (345)	Nkx2			4	9.9 × 10^−5^
rs752045	8	5937538	Intergenic	CSMD1 (1098) LOC100287015 (311)	CEBPB; GR	Skeletal muscle myotubes differentiated from HSMM cell line		12	1
rs2142991	10	42661111	Intergenic	BMS1 (11) LINC01264 (133)	CEBPB; CTCF; Smad4			40	1
rs2796549	10	42686043	Intergenic	BMS1 (36) LINC01264 (108)		Skeletal muscle myoblasts; aortic smooth muscle		1	1
rs3121278	10	42695652	Intergenic	BMS1 (45) LINC01264 (99)	GR	Skeletal muscle myotubes differentiated from HSMM cell line; osteoblasts		35	1
rs7128512	11	73049947	Intronic	PLEKHB1	Roaz		Weak promoter in skeletal muscle myoblasts^1^	3	0.266
rs6590	11	73051200	UTR3	PLEKHB1	NRSF		Enhancer in skeletal muscle^2^; weak enhancer in stomach smooth muscle^2^	15	0.057
rs11235843	11	73051644	Downstream	PLEKHB1	Nrf‐2		Enhancer in skeletal muscle^2^; weak enhancer in stomach smooth muscle^2^	15	0.057

a Enhancer/promoter enrichment in muscle cells including SNPs in linkage disequilibrium with GWAS lead SNPs.

b The change in log‐odds (LOD) scores of regulatory motifs larger than 10 were reported; ^1^Annotation from ENCODE Database; ^2^ Annotation from Epigenetic Roadmap.

c SNPs in LD: Number of SNPs in LD (*r* ^2^ ≥ 0.8 and MAF ≥ 1%, based on 1000 Genome Project) with the lead GWAS SNP in each locus.

d Permutation *P*‐values corrected for multiple testing: This analysis included all SNPs in LD with the GWS lead SNPs. Multiple testing corrected permutation *P*‐values < 0.05 are considered statistically significant.

### eQTL analysis

The top five SNPs listed in Table 1 were queried as index SNP in skeletal muscle and brain tissue eQTL. For the locus on chromosome 10 (rs3121278), a proxy SNP rs3121327 (*r* ^2 ^= 0.87) was significantly associated with gene transcript zinc finger protein 33B (*ZNF33B*) in prefrontal cortex tissue. No other associations were observed for the other loci queried.

## Discussion

The combined discovery and replication meta‐analysis resulted in increased significance in the chr8p23 locus, exceeding genomewide significance (rs752045, *P*‐value = 3.18 × 10^−10^ and rs890022, *P*‐value = 4.80 × 10^−8^). We conducted a genomewide association analysis of lower body strength in a smaller sample as a second trait for muscle strength. However, there were no significant genetic associations observed for lower body strength, and the results did not confirm the top signals from the grip strength analysis.

The chromosome 8p23 locus—rs752045—is over 500 kb away from the closest gene genomewide significant association. However, according to the ENCODE's DNase I hypersensitivity data, rs752045 is located in an accessible chromatin region, indicating possible regulatory activities in skeletal muscle myotubes differentiated from the HSMM cell line. This SNP alters a binding motif of the CCAAT/enhancer‐binding protein beta (CEBPB). The effect allele (G) decreases a score developed to define the effect of variants on regulatory motifs (the position weight matrix (PWM) score). In this case, the PWM score for CEBPB decreased from 11.6 to −0.2, indicating a prediction of decreased binding affinity of CEBPB. The PWM scores were reported as part of the HaploReg database (http://www.broadinstitute.org/mammals/haploreg/detail_v4.1.php?query=%26id=rs752045). CEBPB is a transcription factor that regulates genes for inflammatory responses, including the IL‐1 response element in the *IL‐6* gene (Harries *et al*., 2012). IL‐6 levels are strongly related to muscle strength, functional decline, and sarcopenia in older adults (Cesari *et al*., 2004; Kilgour *et al*., 2013). CEBPB is also important in macrophage function, which plays a crucial role in normal skeletal muscle repair (Rahman *et al*., 2012). In addition, expression of CEBPB in blood leukocytes has been positively associated with muscle strength in humans, further supporting the possible link between gene variants and a decline in skeletal muscle function in older age groups (Ruffell *et al*., 2009).

SNPs in associated regions on chromosomes 7 and 11 are proximal to genes *PLEKHB1* (chr11), *FAM3C* (chr7), and *WNT16* (chr7), and the latter has been associated with bone mineral density, osteoporosis, and fracture risk. Both loci represent promoters or enhancers in regulatory chromatin states in skeletal muscle myoblasts in ENCODE and Epigenetic Roadmap data. PLEKHB1 protein interacts with ACVR1, which is involved in fibrodysplasia ossificans progressiva (FOP), a rare congenital disorder that causes bone formation in muscles, tendons, ligaments, and connective tissues. Additionally, SNPs on the chromosome 7 locus were predicted to alter binding motifs of the CCAAT/enhancer‐binding protein beta, delta, and gamma family (CEBPB, CEBPD, and CEBPG) and the nuclear factor kappa‐β (NF‐kB). In addition to the *CEBPB* association with muscle discussed above, *CEBPD* has also been linked to differential expression of myostatin, a skeletal muscle inhibitory factor that can lead to muscle strength declines (Allen *et al*., 2010). CEBPG likely plays a role in cell growth arrest in the setting of inflammation activation (Huggins *et al*., 2013). NF‐kB is the nuclear transcription factor that acts as a gate‐keeping molecule for activation of inflammatory signaling (Guttridge *et al*., 2000; Ershler, 2007). Subtle alteration in expression of these factors may well alter muscle tissue maintenance with aging and would in turn lead to grip strength declines.

Last, the suggestive region of chromosome 10 is 20 kb away from the *BMS1L* gene, a ribosome assembly protein which has no known function in skeletal muscle. This group of three SNPs also had relevant data from ENCODE, indicating that DNase‐hypersensitive sites were found in skeletal muscle myotubes, in particular those differentiated from HSMM cell lines and osteoblasts.

There are several strengths to this study. First, we have identified 14 cohorts including 27 581 older adults that have appropriate handgrip strength measurements and genotypes necessary to perform a study of this kind. Next, the ability to explore potential findings with the ENCODE data provides an important biologic window into the potential relevance of the genetic findings. There are potential limitations to this study as well. First, a cross‐sectional, one‐time handgrip, or lower body strength measure may not be the best phenotypic measurement to capture age‐related strength decline as a phenotype. Although the lower body strength analysis was consistent with grip strength, due to sample size restrictions, the age cutoff for lower body strength was set at 50 years of age. The correlation between grip and lower body strength has been reported to be in the range of 0.4–0.6, suggesting that both measure the same construct of muscle strength (Bohannon *et al*., 2012).

This cross‐sectional study was designed to determine genetic variants associated with grip strength in persons over the age of 65 years. Strength in old age is thought to be a reflection of both the peak strength and the rate of decline. Similarly, cross‐sectional analysis with phenotypes such as bone density or cognitive performance still have been useful for understanding rate of decline with age. Here, we studied individuals over 65 years of age; thus, the majority are predicted to have already entered the decline phase. Future genetic studies should consider examining changes in muscle strength to focus on the potential determinants of age‐related decreases that are commonly observed with aging, as trajectories of strength decline were not widely available among these cohorts.

Despite limitations, these results suggest biologically plausibility. Chromosome 7 locus was significantly enriched for enhancer/promoter elements in muscle cells compared with other muscle types. C/EBP transcription factors have been linked to a number of metabolic and inflammatory processes that would be expected to influence skeletal muscle, and have been previously implicated in other cohorts. These findings provide additional rationale for the further study of C/EBP‐related pathways and their overall influence in the development of dynapenia in older adults. Future studies should follow up these findings to determine whether there are potential epigenetic changes, or even whether there are significant CEBPB expression differences in skeletal muscle samples between young and old humans.

## Experimental procedures

## Subjects

The discovery phase of this GWAS was conducted on 27 581 subjects from the following 14 participating studies of the Cohorts for Heart and Aging Research in Genomic Epidemiology Consortium (CHARGE); the Age, Gene/Environment Susceptibility Study (AGES); the Cardiovascular Health Study (CHS); the Framingham Heart Study (FHS); the Health, Aging, and Body Composition (Health ABC) Study; the Health and Retirement Study (HRS); the InCHIANTI Study; the Lothian Birth Cohort Studies (1921 and 1936); the Osteoporotic Fractures in Men Study (MrOS); Religious Order Study, Memory and Aging Project (MAP/ROS); the Study of Health in Pomerania (SHIP); the Study of Osteoporotic Fractures (SOF); the Tasmanian Study of Cognition and Gait (TasCog); and the Twins UK Study. Replication cohorts contributed 6393 subjects from three cohorts, the Atherosclerosis Risk in Communities Study (ARIC) and the Rotterdam Studies I and II. Detailed description of each cohort and references are included in the Appendix S1 (Supporting information). Each cohort's study protocol was reviewed and approved by their respective institutional review board.

In parallel to grip strength analysis, a GWAS analysis of lower body strength was conducted as an additional measure of muscle strength in 9822 individuals over the age of 50 years from seven studies: AGES, Baltimore Longitudinal Study on Aging (BLSA), InCHIANTI, CHS, FHS, Health ABC, and MAP/ROS.

### Phenotyping

All participants with at least one recorded grip strength measurement (kg) (Table S1) were included in the analysis. The primary outcome was defined as the maximal value across available trials. Exclusion criteria for grip strength analysis included age <65 years, non‐Caucasian origin via self‐report or identical‐by‐state (IBS) clustering of the GWAS data, and missing grip strength data. Additional exclusion based on self‐reported pain, surgery, or osteoarthritis in the dominant hand was considered. However, as adequate data across all cohorts were not available, these exclusions were not implemented in this analysis. Handgrip was employed as a nontransformed, continuous trait.

For lower body strength, all studies used performance‐based assessment methods reporting measures in kg or in Newton‐meter (Table S2). If multiple examinations were performed, the maximum measurement was used. Exclusion for lower body strength analysis was consistent with grip strength; however, due to sample size restrictions, the age cutoff was set at 50 years of age. Lower leg strength was analyzed as a nontransformed, continuous trait.

Additional variables used in this study included gender, age, standing height, and weight for both grip and lower body strength. Each of these characteristics was collected with handgrip and/or lower body strength according to study‐specific protocols.

### Genotyping

Each cohort performed its own genomewide genotyping and genotype imputation based on NCBI Build 36 (http://www.ncbi.nlm.nih.gov/SNP/). Table S3 (Supporting information) summarizes genotyping platform, imputation methods, quality control methods, and final SNP count per cohort. Results are reported for each SNP for as many cohorts as were available via genotyping and imputation.

### Statistical analysis

Multiple linear regression models were built for genotyped and imputed SNPs on maximal grip strength (kg), adjusted for age, gender, height, weight, study site (when necessary), and principal components to control for population stratification (Price *et al*., 2006). An additive model with the count of the number of variant alleles was used for all analyses. Handgrip strength was used as a continuous trait, and the regression results reflect an increase or decrease in strength (kg) per additive allele. Test statistics for genomewide association analysis were combined using METAL (Willer *et al*., 2010). Inverse variance‐weighted meta‐analysis was performed using a fixed‐effects model of β‐estimates and standard errors from each cohort. In the meta‐analysis of discovery GWAS, between‐study heterogeneity was tested using Cochran's Q test as implemented in METAL. A threshold of *P*‐value <5 × 10^−8^ was utilized to determine genomewide statistical significance, while *P*‐values <1 × 10^−5^ were considered suggestive. SNPs that met these significance thresholds were then evaluated in a set of 3 replications cohorts, as well as analyzed jointly in discovery and replication cohorts (*n* = 33 974).

For the leg strength analysis, as the unit of measure differed by cohort (kg or Nm), a sample size‐weighted meta‐analysis was conducted where an arbitrary reference allele is selected and a z‐statistic summarizing the magnitude and the direction of effect relative to the reference allele was calculated and weighted by the square root of the sample size of each study. Thresholds for statistical significance set for the handgrip analysis were utilized for the leg strength results as well.

Using the HaploReg tool (http://compbio.mit.edu/HaploReg.), we annotated potential regulatory functions of our GWAS SNPs and loci based on experimental epigenetic data, including open chromatin and histone modifications, and transcription factor binding sites in human cell lines and tissues (Ward & Kellis, 2012). First, we constructed haplotype blocks for GWAS most significant, or lead, SNPs and SNPs in high linkage disequilibrium (LD, *r* ^2^ > 0.8) with GWAS lead SNPs. Then, we identified regulatory elements including enhancers and promoters estimated by chromatin states in the haplotype blocks across 98 healthy human tissues/normal cell lines available in the ENCODE Project and the Epigenomics Roadmap Project (Encode and Consortium 2011; Chadwick, 2012). The regulatory elements were annotated by an algorithm named ChromHMM, and data were downloaded from HaploReg3 (Ernst & Kellis, 2012; Ward & Kellis, 2012). To evaluate whether GWAS loci were enriched with regulatory elements and corresponded to the DNase I‐hypersensitive sites (DHSs) in muscle tissues, we performed a promoter/enhancer enrichment analysis using a hypergeometric test to compare the abundance of regulatory elements in muscle tissues (9 relevant muscle tissues/cell lines) to nonmuscle tissues (89 tissues/cell lines) in the haplotype blocks of a GWAS locus. A permutation was performed to correct for multiple testing. Permutation *P*‐values <0.05 were considered statistically significant.

### Expression quantitative trait loci (eQTL) analysis

Proxy SNPs in linkage disequilibrium (*r* ^2^>0.8) in European ancestry populations were identified for handgrip for the top five most significant SNPs as the lead SNPs using SNAP (Johnson *et al*., 2008). Index SNPs and proxies were identified in a collected database of expression SNP (eSNP) results. The collected eSNP results met criteria for statistical thresholds for association with gene transcript levels as described in the original papers. A general overview of a subset of >50 eQTL studies has been published (Zhang *et al*., 2014), with specific citations for >100 studies. For the current query, we focused our search to skeletal muscle and brain tissue (Keildson *et al*., 2014; Zhang *et al*., 2014). Details on tissue samples can be found in the Appendix S1 (Supporting information).

## Funding

Johns Hopkins University Claude D. Pepper Older Americans Independence Center (NIA Grant/Award Number P30 AG021334), Wellcome Trust, (Grant/Award Number: ‘FP7/2007–2013’) National Institute for Health Research, National Institute of Diabetes and Digestive and Kidney Diseases, (Grant/Award Number: ‘DK063491’) German Federal Ministry of Education and Research, (Grant/Award Number: ‘01ZZ0103’,’01ZZ0403’,’01ZZ9603’,’03IS2061A’,’03ZIK012’), National Institutes of Health, (Grant/Award Number: ‘HHSN268200625226C’,’HHSN268200782096C’,’N01AG12100’,’UL1RR025005’), Biotechnology and Biological Sciences Research Council, National Health and Medical Research Council, (Grant/Award Number: ‘NHMRC100089’,’NHMRC1034483’,’NHMRC1061457’,’NHMRC403000’,’NHMRC491109’,’NHMRC606543’), National Institute on Aging, (Grant/Award Number: ‘1R01AG032098‐01A1 ‘,’263 MD 821336’,’263 MD 9164 ‘,’AG016495’,’AG033193’,’AG08122’,’N01AG62101’,’N01AG62103’,’N01AG62106’,’P30AG10161’,’R01 AG005394’,’R01 AG005407’,’R01 AG027574’,’R01 AG027576’,’R01 AR35582’,’R01 AR35583’,’R01AG023629’,’R01AG040039’,’R01AG15819’,’R01AG17917’,’R01AG24480’,’R01AG29451’,’R01AG30146’,’R01AR35584’,’RC2 AG036495’,’U01 AG18197’,’U01‐AG027810’,’U01AG009740’), National Institute of Arthritis and Musculoskeletal and Skin Diseases, (Grant/Award Number: ‘R01 AR41398’,’R01‐AR051124’,’RC2ARO58973’,’U01 AR45580’,’U01 AR45583’,’U01 AR45614’,’U01 AR45632’,’U01 AR45647’,’U01 AR45654’) National Heart, Lung, and Blood Institute, (Grant/Award Number: ‘HHSN268200800007C’,’HHSN268201100005C’,’HHSN268201100006C’,’HHSN268201100007C’,’HHSN268201100008C’,’HHSN268201100009C’,’HHSN268201100010C’,’HHSN268201100011C’,’HHSN268201100012C’,’HHSN268201200036C’,’N01HC25195’,’N01HC55222’,’N01HC85079’,’N01HC85080’,’N01HC85081’,’N01HC85082’,’R01HL086694’,’R01HL087641’,’R01HL103612’,’R01HL120393’,’R01HL59367’,’U01HL080295’), National Human Genome Research Institute, (Grant/Award Number: ‘U01HG004402’) Medical Research Council, Research Institute for Diseases in the Elderly, (Grant/Award Number: ‘014‐93‐015’,’RIDE2’) Netherlands Organization for Scientific Research NWO Investments, (Grant/Award Number: ‘050‐060‐810’,’175.010.2005.011’,’911‐03‐012’), National Center for Research Resources, (Grant/Award Number: ‘UL1 RR024140’). “N02HL64278“, “N01‐AG‐1‐2100“, “HHSN27120120022C“, “HL105756“, “N01HC85083“, “N01HC85086“, “RC1 AG035835“, NIA Intramural Research Program, Hjartavernd (the Icelandic Heart Association), the Althingi (the Icelandic Parliament), Research Foundation Flanders (FWO), UK Biotechnology and Biological Sciences Research Council, The Royal Society, The Chief Scientist Office of the Scottish Government, Age UK (The Disconnected Mind project), Lifelong Health and Wellbeing Initiative: “MR/K026992/1“, Medical Research Council, Netherlands Organization of Scientific Research NWO Investments: “nr.“175.010.2005.011“, “911‐03‐012“, “050‐060‐810“, Research Institute for Diseases in the Elderly: “014‐93‐015“; “RIDE2“, Netherlands Genomics Initiative (NGI)/Netherlands Organisation for Scientific Research (NWO) Netherlands Consortium for Healthy Aging (NCHA): “nr. 050‐060‐810.“

## Conflict of interest

There are no additional conflict of interests.

## Author contributions

All authors were involved in data collection, study design, development of methods, and review and final approval of the manuscript. In addition, AMM, TT, WCC, JDE, ADJ, AMA, MLC, GD, DSE, BH, KL, KLL, MM, AVS, JAS, AT, and LY, DEA, ASB, AH, YH, FR, AU were involved in data analysis. AMM, TT, DK, GA, WCC, ADE, ADJ, ABN, JDW, DPK, and JMM were responsible for writing the manuscript.

## Supporting information


**Fig. S1** Quantile‐Quantile plot of expected vs. observed –log10 *P*‐values for meta‐analysis of genome‐wide association of grip strength.
***Fig. S2** Genome‐wide scans of grip strength of CHARGE cohorts.*
**Fig. S3** Quantile‐Quantile plot of expected vs. observed –log10 *P*‐values for meta‐analysis of genome‐wide association of leg strength

**Table S1** Details of Hand Grip Measure Collection per Cohort
**Table S2** Assessment methods and cohort descriptive for lower leg strength analysis
**Table S3** Genotyping and Data Cleaning Details per Discovery Cohort
**Table S4** Top SNPs from the meta‐analysis of grip strength genome‐wide associations in 14 discovery cohorts
**Table S5** Most significant non‐redundant association from meta‐analysis of lower body strength in 9822 individuals
**Table S6** Associations from meta‐analysis of lower body strength for the top signals from the grip strength meta‐analysis.

**Appendix S1** Detailed Description of Discovery Cohorts
