# Supplementary material for: GWAS analysis of handgrip and lower body strength in older adults in the CHARGE consortium
Source: Aging Cell. 2016 Jun 21;15(5):792–800. doi: 10.1111/acel.12468 (PMC5013019; doi:10.1111/acel.12468)
Supplement: Supplementary file 1 — Fig. S1 Quantile‐Quantile plot of expected vs. observed –log10 P‐values for meta‐analysis of genome‐wide association of grip strength. Fig. S2 Genome‐wide scans of grip strength of CHARGE cohorts. Fig. S3 Quantile‐Quantile plot of expected vs. observed –log10 P‐values for meta‐analysis of genome‐wide association of leg strength [file ACEL-15-792-s001.docx]

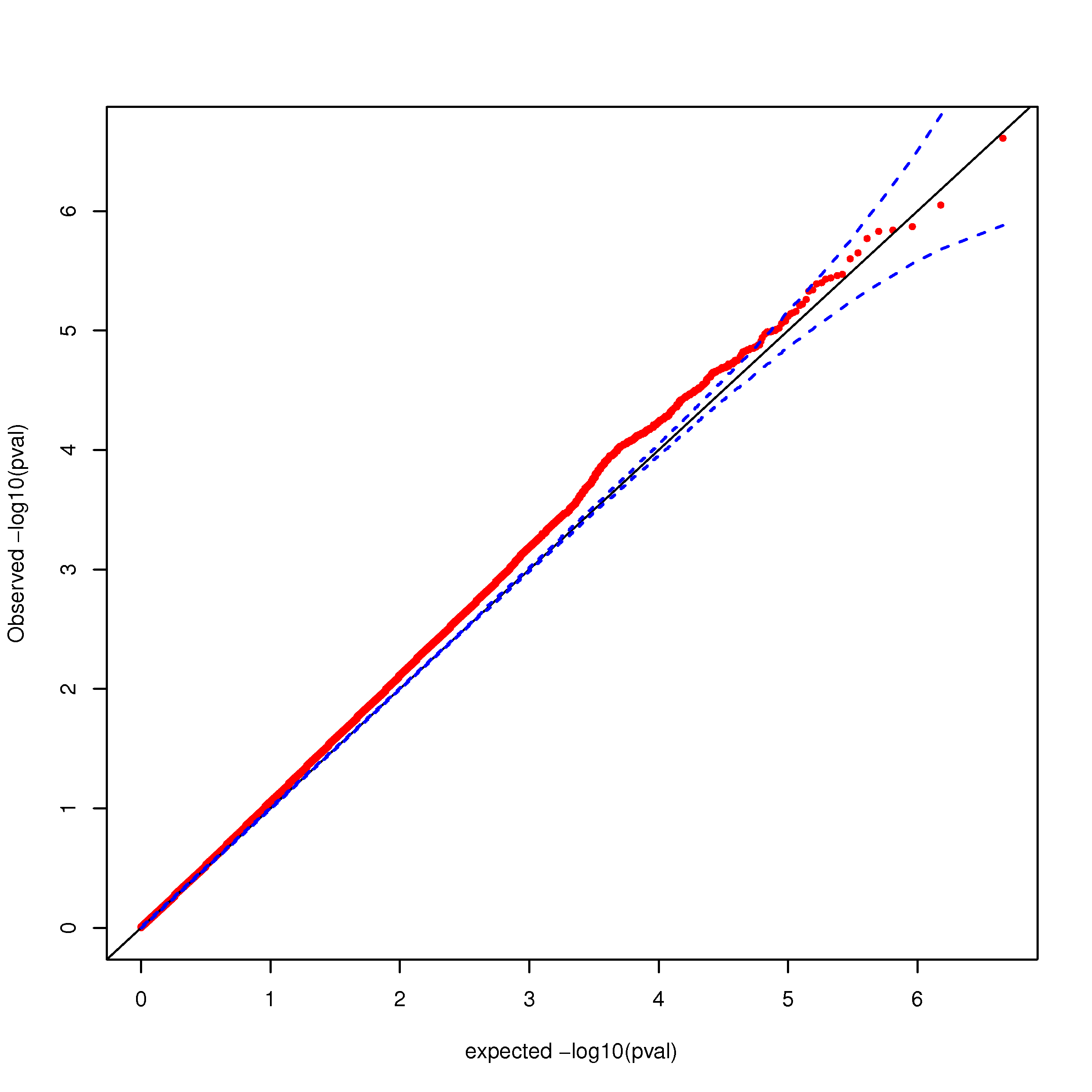


**Supplemental Figure S1**. Quantile-Quantile plot of expected versus observed –log10 p-values for meta-analysis of genome-wide association of grip strength.


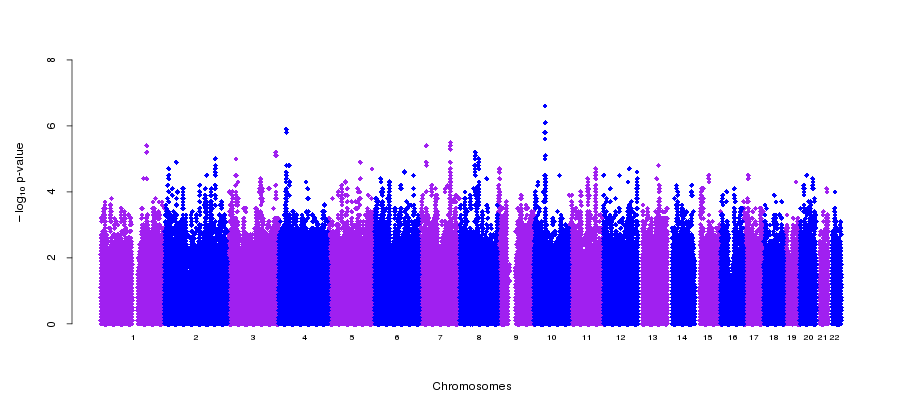


**Supplemental Figure S2**. Genome-wide scans of grip strength of CHARGE cohorts. Genome-wide associations of grip strength for ~2.5 million imputed and genotyped HapMap SNPs.


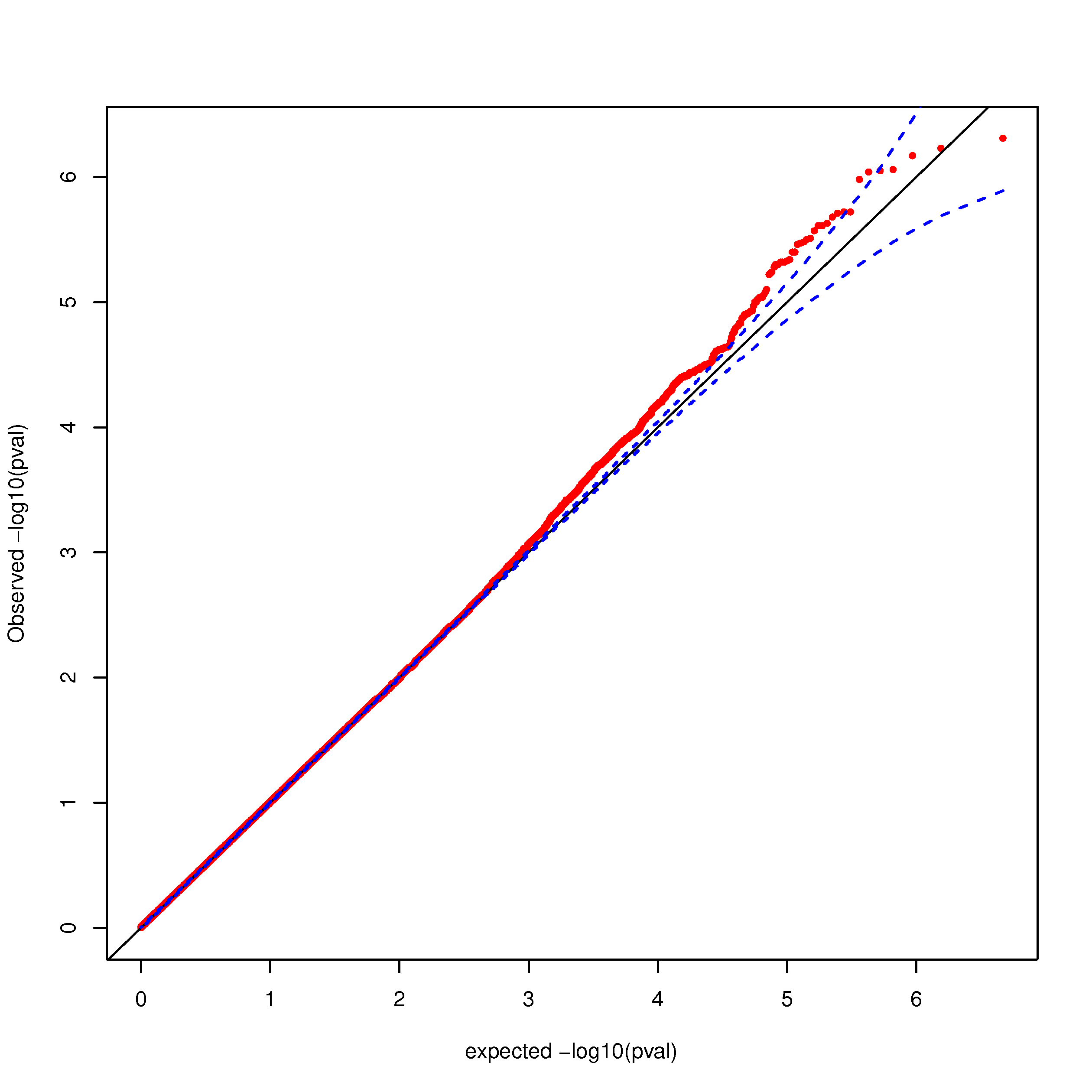


**Supplemental Figure S3.** Quantile-Quantile plot of expected versus observed –log10 p-values for meta-analysis of genome-wide association of leg strength.
