# Supplementary material for: GWAS analysis of handgrip and lower body strength in older adults in the CHARGE consortium
Source: Aging Cell. 2016 Jun 21;15(5):792–800. doi: 10.1111/acel.12468 (PMC5013019; doi:10.1111/acel.12468)
Supplement: Supplementary file 2 — Table S1 Details of Hand Grip Measure Collection per Cohort Table S2 Assessment methods and cohort descriptive for lower leg strength analysis Table S3 Genotyping and Data Cleaning Details per Discovery Cohort Table S4 Top SNPs from the meta‐analysis of grip strength genome‐wide associations in 14 discovery cohorts Table S5 Most significant non‐redundant association from meta‐analysis of lower body strength in 9822 individuals Table S6 Associations from meta‐analysis of lower body strength for the top signals from the grip strength meta‐analysis. [file ACEL-15-792-s002.docx]

**Supplemental Tables**

**Supplemental Table S1. Details of Hand Grip Measure Collection per Cohort**

| **Study** | **N** | **N Male**  **(%)** | **Mean Age years ± SD** | **Mean grip, kg ± SD** | **Hand Grip Assessment Description** |
| --- | --- | --- | --- | --- | --- |
| **AGES** | 2,981 | 1250 (41.9) | 76.4**±**5.5 | 30.3**±**10.7 | Good Strength computerized dynamometer, three trials per hand and Jamar hydraulic hand dynamometer, two trials per hand |
| **ARIC** | 3,714 | 1,654 (44.5) | 75.8**±**5.1 | 28.3**±**10.4 | Jamar hydraulic hand dynamometer, two trials per hand |
| **CHS** | 3052 | 1203  (39.4) | 72.3**±**5.3 | 29.0±10.5 | Jamar hydraulic hand dynamometer, three trials per hand |
| **FHS** | 1,540 | 678 (44.0) | 74.6**±**7.0 | 27.1**±**11.2 | Jamar hydraulic hand dynamometer, three trials per hand |
| **HealthABC** | 1,636 | 868 (53.1) | 73.8**±**2.8 | 32.2**±**10.4 | Jamar hydraulic hand dynamometer, two trials per hand |
| **HRS** | 5,178 | 2294  (44.3) | 74.6 | 29.6**±**10.5 | Smedley spring-type handgrip dynamometer, two trials per hand |
| **InChianti** | 786 | 348 (44.3) | 74.5**±**6.9 | 28.8**±**12.0 | Jamar hydraulic hand dynamometer, two trials per hand |
| **LBC1921** | 512 | 211  (41.2) | 79.0**±**0.6 | 26.4**±**9.0 | Jamar hydraulic hand dynamometer, three trials per hand |
| **LBC1936** | 1,002 | 509  (50.8) | 69.5**±**0.8 | 29.8**±**10.2 | Jamar hydraulic hand dynamometer, three trials per hand |
| **MAP/ROS** | 1,597 | 481 (30.1) | 79.0**±**7.3 | 25.7**±**10.2 | Jamar hydraulic hand dynamometer, two trials per hand |
| **MrOS** | 4,489 | 4489 (100) | 74.0**±**6.0 | 41.5**±**8.5 | Jamar hydraulic hand dynamometer, two trials per hand |
| **RSI** | 1,441 | 587 (40.7) | 79.1**±**4.8 | 23.5**±**9.0 | Jamar hydraulic hand dynamometer, three trials per hand |
| **RSII** | 1,238 | 561 (45.3) | 72.0**±**5.2 | 27.0**±**9.6 | Jamar hydraulic hand dynamometer, three trials per hand |
| **SHIP** | 320 | 166 (51.9) | 72.2**±**5.6 | 32.5**±**11.2 | Jamar hydraulic hand dynamometer, one trial per hand |
| **SOF** | 3,407 | 0 (0) | 73.5**±**5.3 | 20.4**±**4.8 | Jamar hydraulic hand dynamometer, two trials per hand |
| **TasCog** | 371 | 217  (58.5) | 72.2**±**7.0 | 9.2**±**2.8* | North Coast bulb dynamometer, two trials per hand |
| **Twins UK** | 710 | 0  (0) | 70.6**±**4.2 | 25.2**±**6.0 | Jamar hydraulic hand dynamometer, two trials per hand |

***TasCog study used North Coast bulb dynamometer which yielded units of pounds per square inch (PSI).**

**Supplemental Table S2. Assessment methods and cohort descriptive for lower leg strength analysis.**

| **Study** | **N** | **Age+SD** | **Leg Strength Unit** | **Leg Strength Mean**+**SD** | **Leg Strength Assessment Description** |
| --- | --- | --- | --- | --- | --- |
| AGES | 3,004 | 76.4+5.4 | Nm | 317.7+115.2 | Maximal isometric extension force of the knee is tested in a strain gauge transducer in 60° flexion; exclude subjects with recent leg surgery or history of ischemic heart conditions. |
| BLSA | 529 | 69.5 + 14.1 | Nm | 114.2+47.1 | This is concentric torque measured at 30d/s. We also have 180d/s and eccentric torque measured at same speed. We also have (isometric) peak torque at 120° and 140°. Exclusion include pain, knee replacement, osteoporosis, nonmedicated high blood pressure >220/115 |
| InCHIANTI | 943 | 67.2+15.3 | kg | 17.5+6.8 | Maximal voluntary isometric strength of knee extensors was measured using a hand-held dynamometer (Nicholas Muscle Tester; Sammon Preston Inc., Chicago, IL, USA) according to a standard assessment protocol (test–retest reliability 0.85, inter-rater reliability 0.74). The test was repeated three times per leg |
| CHS | 638 | 80+3.60 | Nm | 69.03 + 36.05 | The isometric chair is used to measure strength with knee extension, and was designed for the Health ABC study. Average of 2 tests on right leg. Exclude subjects with severe movement limitation or pain. The middle of the participant’s thigh to be at the end of the chair with their knee joint at a 90-degree angle. |
| FHS | 2,388 | 60.7 ± 9.3 | kg | 20.1 ± 6.5 | Quadriceps strength of the right leg was measured using a Nicholas hand-held isometric dynamometer. In cases where the right leg could not be assessed (e.g. painful, leg brace), the left leg was measured. In a seated position with hands on lap and back supported against the chair back, the participants’ right knee was positioned at 60 degrees of flexion using a fixed goniometer with the right foot placed flat on the floor. The dynamometer was held perpendicular to the leg on the anterior surface of the tibia 6 cm above the lateral malleolus and the participant was instructed to kick their leg against the dynamometer as hard as they could for 3 seconds. The single tester for all subjects was able to place her back against an upright object to minimize the chance of leg extension pushing the examiner backwards. The procedure was repeated and the force (kg) for both trials was recorded. |
| HABC | 1,460 | 73.7 ± 2.8 | Nm | 106.9 ± 38.2 | Quadriceps strength was measured using an isokinetic dynamometer (Kin-Com dynamometer, 125 AP) for knee extension |
| ROS/MAP | 860 | 81.1 ± 6.5 | Nm | 10.9 ± 4.3 | Maximal voluntary isometric strength of knee extensors was measured using a hand-held dynamometer ( Kin-Com dynamometer, 125 AP). |

**Supplemental Table S3. Genotyping and Data Cleaning Details per Discovery Cohort**

|  | **AGES** | **CHS** | **FHS** | **HABC** | **HRS** | **InCHIANTI** |
| --- | --- | --- | --- | --- | --- | --- |
| **Study** | Age, Gene/Environment Susceptibility Study | Cardiovascular Health Study | Framingham Heart Study | Health, Aging and Body Composition Study | Health and Retirement Study | The InCHIANTI Study |
| **Design Papers** | PMID:17351290 | PMID:1669507 | PMID: 14819398; 474565 | PMID: 10865790 | PMID: 24671021 | PMID: 11129752 |
| **Array** | Illumina HumanCNV370-Duo Bead Chip | Illumina 370 CNV | Affymetrix Gene Chip 500K Array Set & 50K Human Gene Focused Panel | Illumina Human 1M-Duo | Illumina Omni2.5 Beadchip | Illumina Infinium HumanHap550 |
| **Calling Algorithm** | Bead Studio | Bead Studio | BRLMM | Bead Studio | Bead Studio | Bead Studio |
| **Per SNP Call Rate** | <97% | <97% | <97% | <97% | <98% | <99% |
| **HWE p-value** | <10^-5^ | <10^-5^ | <10^-6^ | <10-6 | <10^-4^ | <10^-4^ |
| **Mendellian errors** | NA | ≤2 | N>100 | NA | ≤2 | NA |
| **Excess heterozygosity** | NA | ND | subject heterozygosity >5 SD away from mean | ND | NA | NA |
| **MAF** | <1% | Excluded SNPs with 0 heterozygotes | <1% | <1% | <0.5% | <1% |
| **Number of SNPs used for imputation** | 308,340 | 306,655 | 385,958 | 914,263 | 2,509,413 | 498,838 |
| **Imputation software** | Mach1v1.0.16 | BIMBAM | Mach1v1.0.15 | MACH v.1.0.16 | Mach1v1.0.16 | Mach |
| **Imputation backbone/ NCBI Build** | Build 36 | Build 36 | Build 36 | Build 36 | Build 36 | Build 36 |
| **GWAS Statistical Analysis** | ProbABEL 0.0.6, R | R | R | R | PLINK, R | Merlinoffline |
| **Total number used in the analysis (MAF>0.005)** | 2,408,991 | 2,332,998 | 2,543,887 | 2,513,421 | 4,930,728 | 2,461,088 |
| **Inflation factor (λ)**  **(hand grip, leg strength)** | 1.03, 1.05 | 1.05, 1.05 | 1.03, 1.00 | 1.01, NA | 1.01, NA | 1.01, 1.00 |

**Supplemental Table S3. Genotyping and Data Cleaning Details per Discovery Cohort cont’d**

|  | **LBC 1921** | **LBC 1936** | **MrOS** | **ROS/MAP** | **SHIP** | **SOF** |
| --- | --- | --- | --- | --- | --- | --- |
| **Study** | Lothian Birth Cohort (1921) | Lothian Birth Cohort (1936) | Osteoporotic Fractures in Men Study | Rush Aging and Memory/ Religious Orders Study | The Study of Health in Populations | The Study of Osteoporotic Fractures |
| **Design Papers** | PMID: 14717632 | PMID: 18053258 | PMID: 16085466, 16084776 | PMID:16103727; 22471860 | PMID:20167617 | PMID: 2404146 |
| **Array** | Illumina Human 610_Quadv1 | Illumina Human 610_Quadv1 | Illumina HumanOmni1 Quad | Affymetrix 6.0 | Affymetrix 6.0 | Illumina HumanOmni1 Quad |
| **Calling Algorithm** | Genome Studio | Genome Studio | Bead Studio | Bead Studio | Birdseed2 | Bead Studio |
| **Per SNP Call Rate** | <98% | <98% | <97% | <95% | <98% | <97% |
| **HWE p-value** | <10^-3^ | <10^-3^ | <10-4 | <10^-6^ | ND | <10-4 |
| **Mendellian errors** | NA | NA | NA | NA | ND | NA |
| **Excess heterozygosity** | NA | NA | ND | NA | ND | ND |
| **MAF** | <1% | <1% | <1% | <1% | ND | <1% |
| **Number of SNPs used for imputation** | 542,040 | 542,040 | 740,713 | 645,349 | 869,224 | 740,713 |
| **Imputation software** | Mach1v1.0.16 | Mach1v1.0.16 | Mach1v1.0.16 | Mach1v1.0.16a | IMPUTEv0.5.0 against HapMap II CEU v22 | Mach1v1.0.16 |
| **Imputation backbone/ NCBI Build** | Build 36 | Build 36 | Build 36 | Build 36 | Build 36 | Build 36 |
| **GWAS Statistical Analysis** | ProbABEL | ProbABEL | R | ProbABEL, Plink, R | QuickTestv0.95 | R |
| **Total number used in the analysis (MAF>0.005)** | 2,543,887 | 2,543,887 | 2,461,033 | 2,492,553 | 2,748,910 | 2,411,023 |
| **Inflation factor (λ)**  **(hand grip, leg strength)** | 1.00, NA | 1.00, NA | 1.02, NA | 1.02, 1.01 | 1.02, NA | 1.03, NA |

**Supplemental Table S3. Genotyping and Data Cleaning Details per Discovery Cohort cont’d**

|  | **TasCog** | **TwinsUK** | **BLSA** |
| --- | --- | --- | --- |
| **Study** | Tasmanian Study of Cognition and Gait | The UK Adult Twin Registry | Baltimore Longitudinal Study on Aging |
| **Design Papers** | PMID 18314452 | PMID:23088889 | NIH Publication No. 84-2450, November 1984 |
| **Array** | Illumina Hap370CNV | Illumina HumanHap300 and HumanHap610Q | Illumina 550K |
| **Calling Algorithm** | Illumina GenCall | Illluminus | Bead Studio |
| **Per SNP Call Rate** | <97% | <97% | <99% |
| **HWE p-value** | <10^-7^ | <10^-6^ | <10-4 |
| **Mendellian errors** | NA | NA | NA |
| **Excess heterozygosity** | NA | subject heterozygosity >2 SD away from mean | NA |
| **MAF** | <0.5% | <1% | <1% |
| **Number of SNPs used for imputation** | 313,702 | HumanHap300: 303,940, HumanHap610Q: 553,487 | 514,027 |
| **Imputation software** | MACHv1.0.16 | IMPUTEv0.5.0 against HapMap II CEU v22 | Mach1v1.0.16 |
| **Imputation backbone/ NCBI Build** | Build 36 | Build 36 | Build 36 |
| **GWAS Statistical Analysis** | R | GenABEL | Merlin-offline |
| **Total number used in the analysis (MAF>0.005)** | 2,543,149 | 2,463,208 | 2,543,887 |
| **Inflation factor (λ)**  **(hand grip, leg strength)** | 1.00, NA | 1.01, NA | NA, 1.00 |

**Supplemental Table S4. Top SNPs from the meta-analysis of grip strength genome-wide associations in 14 discovery cohorts.**

| **SNP** | **Chr** | **Position** | **Nearest Gene** | **Effect/Other Allele** | **Frequency Effect Allele** | **Beta (SE)** | **P-value** | *I^2^* | **HetP** |
| --- | --- | --- | --- | --- | --- | --- | --- | --- | --- |
| rs3121278 | 10 | 42695652 | *BMS1L* | T/G | 0.18 | -0.39 (0.07) | 2.68E-08 | 17% | 0.27 |
| rs752045 | 8 | 5937538 | *ANGPT2* | G/A | 0.18 | 0.47 (0.09) | 3.09E-08 | 0% | 0.74 |
| rs3121324 | 10 | 42699521 | *BMS1L* | T/C | 0.19 | -0.34 (0.07) | 2.06E-07 | 17% | 0.28 |
| rs2796549 | 10 | 42686043 | *BMS1L* | A/G | 0.17 | -0.36 (0.07) | 3.29E-07 | 21% | 0.24 |
| rs2795508 | 10 | 42673561 | *BMS1L* | T/G | 0.17 | -0.35 (0.07) | 3.98E-07 | 20% | 0.25 |
| rs3121323 | 10 | 42697172 | *BMS1L* | A/G | 0.17 | -0.34 (0.07) | 5.24E-07 | 20% | 0.24 |
| rs2142991 | 10 | 42661111 | *BMS1L* | C/T | 0.17 | -0.35 (0.07) | 5.71E-07 | 21% | 0.24 |
| rs1819054 | 7 | 1.21E+08 | *FAM3C* | G/A | 0.4 | 0.27 (0.06) | 8.23E-07 | 0% | 0.75 |
| rs1528351 | 7 | 1.21E+08 | *FAM3C* | T/C | 0.42 | 0.26 (0.05) | 1.18E-06 | 0% | 0.61 |
| rs1528353 | 7 | 1.21E+08 | *FAM3C* | A/C | 0.42 | 0.25 (0.05) | 1.43E-06 | 0% | 0.61 |
| rs890022 | 8 | 5927003 | *ANGPT2* | A/G | 0.12 | 0.48 (0.10) | 1.78E-06 | 0% | 0.93 |
| rs10259325 | 7 | 1.21E+08 | *FAM3C* | C/T | 0.48 | -0.25 (0.05) | 1.93E-06 | 0% | 0.90 |
| rs12535189 | 7 | 1.21E+08 | *FAM3C* | C/T | 0.49 | 0.25 (0.05) | 1.99E-06 | 0% | 0.66 |
| rs4731028 | 7 | 1.21E+08 | *FAM3C* | T/A | 0.42 | 0.28 (0.06) | 2.39E-06 | 0% | 0.56 |
| rs1508086 | 8 | 57980052 | *IMPAD1* | T/C | 0.44 | 0.25 (0.05) | 2.71E-06 | 0% | 0.62 |
| rs1876496 | 8 | 57978123 | *IMPAD1* | G/A | 0.45 | 0.25 (0.05) | 3.04E-06 | 0% | 0.72 |
| rs985942 | 8 | 57984794 | *IMPAD1* | T/C | 0.43 | 0.25 (0.05) | 3.08E-06 | 0% | 0.51 |
| rs2077626 | 10 | 42730968 | *BMS1L* | G/A | 0.18 | -0.32 (0.07) | 3.42E-06 | 24% | 0.20 |
| rs3121327 | 10 | 42708291 | *BMS1L* | A/G | 0.18 | -0.32 (0.07) | 3.73E-06 | 20% | 0.24 |
| rs1815716 | 10 | 42730206 | *BMS1L* | A/G | 0.18 | -0.32 (0.07) | 4.02E-06 | 28% | 0.17 |
| rs4452553 | 5 | 1.24E+08 | *ZNF608* | A/G | 0.18 | -0.32 (0.07) | 4.25E-06 | 57% | 0.39 |
| rs4626333 | 5 | 1.24E+08 | *ZNF608* | T/C | 0.18 | -0.32 (0.07) | 4.39E-06 | 58% | 0.39 |
| rs1891324 | 10 | 42719587 | *BMS1L* | G/A | 0.18 | -0.31 (0.07) | 4.53E-06 | 27% | 0.18 |
| rs1396122 | 8 | 57987417 | *IMPAD1* | A/G | 0.43 | 0.24 (0.05) | 5.48E-06 | 21% | 0.23 |
| rs2795527 | 10 | 42590246 | *BMS1L* | T/C | 0.15 | -0.34 (0.08) | 5.48E-06 | 10% | 0.34 |
| rs1396123 | 8 | 57987452 | *IMPAD1* | T/C | 0.43 | 0.24 (0.05) | 5.54E-06 | 22% | 0.23 |
| rs10101449 | 8 | 57990051 | *IMPAD1* | C/A | 0.42 | 0.24 (0.05) | 6.56E-06 | 24% | 0.21 |
| rs7017465 | 8 | 57988034 | *IMPAD1* | A/G | 0.43 | 0.24 (0.05) | 6.89E-06 | 22% | 0.23 |
| rs2317795 | 8 | 57978056 | *IMPAD1* | G/C | 0.44 | 0.26 (0.06) | 7.11E-06 | 0% | 0.52 |
| rs6987876 | 8 | 57970497 | *IMPAD1* | A/G | 0.44 | 0.24 (0.05) | 7.12E-06 | 5% | 0.40 |
| rs12681817 | 8 | 57989122 | *IMPAD1* | T/C | 0.42 | 0.24 (0.05) | 7.61E-06 | 31% | 0.14 |
| rs1532972 | 8 | 57976444 | *IMPAD1* | T/A | 0.44 | 0.26 (0.06) | 8.00E-06 | 0% | 0.57 |
| rs1860614 | 12 | 675799 | *NINJ2* | C/T | 0.36 | 0.24 (0.05) | 8.08E-06 | 35% | 0.11 |
| rs6474087 | 8 | 57988374 | *IMPAD1* | G/T | 0.43 | 0.23 (0.05) | 8.70E-06 | 16% | 0.28 |
| rs1354969 | 8 | 57988743 | *IMPAD1* | T/C | 0.42 | 0.23 (0.05) | 8.73E-06 | 27% | 0.18 |
| rs7128512 | 11 | 73049947 | *PLEKHB1* | A/G | 0.15 | -0.33 (0.07) | 8.97E-06 | 0% | 0.52 |
| rs1983496 | 7 | 1.21E+08 | *FAM3C* | A/G | 0.47 | -0.23 (0.05) | 8.99E-06 | 0% | 0.93 |
| rs11235843 | 11 | 73051644 | *PLEKHB1* | A/G | 0.1 | -0.38 (0.08) | 9.23E-06 | 0% | 0.47 |
| rs4737447 | 8 | 57974789 | *IMPAD1* | G/A | 0.44 | 0.24 (0.05) | 9.24E-06 | 0% | 0.44 |
| rs17019384 | 1 | 2.13E+08 | *ATF3* | T/C | 0.05 | 0.62 (0.14) | 9.32E-06 | 0% | 0.74 |
| rs6590 | 11 | 73051200 | *PLEKHB1* | A/G | 0.1 | -0.37 (0.08) | 1.00E-05 | 0% | 0.52 |

**Supplemental Table S5**. Most significant non-redundant association from meta-analysis of lower body strength in 9,822 individuals.

| SNP | Chr | Position | #SNPs in locus | Nearest Gene (50kb) | Effect Allele/ Other Allele | Frequency Effect Allele | Zscore | P-value | *I^2^* | HetP |
| --- | --- | --- | --- | --- | --- | --- | --- | --- | --- | --- |
| rs12135534 | 1 | 231473800 | 6 | PCNXL2 | a/c | 0.13 | 4.76 | 2.35E-06 | 0% | 0.79 |
| rs6710199 | 2 | 6048910 | 1 | LOC150622 | a/t | 0.92 | -4.74 | 2.54E-06 | 7% | 0.20 |
| rs16884514 | 6 | 21223003 | 2 | CDKAL1 | a/t | 0.06 | -4.46 | 9.72E-06 | 0% | 0.50 |
| rs969930 | 8 | 70967579 | 1 | N/A | t/c | 0.63 | 4.97 | 8.21E-07 | 0% | 0.62 |
| rs4980177 | 10 | 124855987 | 2 | ACADSB | a/g | 0.46 | 4.57 | 5.70E-06 | 0% | 0.94 |
| rs16831 | 11 | 126833398 | 10 | N/A | t/c | 0.24 | -5.03 | 6.07E-07 | 0% | 0.60 |
| rs931810 | 13 | 75830823 | 1 | N/A | t/c | 0.08 | -4.43 | 1.13E-05 | 0% | 0.44 |
| rs1689953 | 15 | 99047017 | 4 | ASB7 | t/g | 0.70 | -4.76 | 2.28E-06 | 0% | 0.83 |
| rs3112732 | 16 | 7769309 | 1 | N/A | t/g | 0.13 | 4.58 | 5.48E-06 | 0% | 0.30 |
| rs2671668 | 17 | 44894021 | 1 | PHB, NGFR | a/t | 0.27 | -4.46 | 9.46E-06 | 0% | 0.89 |
| rs11867552 | 17 | 74683490 | 1 | HRNBP3 | t/c | 0.28 | -4.54 | 6.75E-06 | 10% | 0.18 |
| rs466850 | 21 | 13720108 | 9 | N/A | c/g | 0.29 | 4.72 | 2.80E-06 | 0% | 0.56 |
| rs12330067 | 22 | 23755439 | 1 | KIAA1671 | a/c | 0.58 | 4.76 | 2.28E-06 | 0% | 0.50 |

Adjusted for genomic control (λ)= 1.02

**Supplemental Table S6**. Associations from meta-analysis of lower body strength for the top signals from the grip strength meta-analysis.

| SNP | Chr | Position | Effect Allele/ Other Allele | Frequency Effect Allele | Zscore | P-value | *I^2^* | HetP |
| --- | --- | --- | --- | --- | --- | --- | --- | --- |
| rs3857836 | 7 | 120931488 | a/g | 0.47 | -1.16 | 2.49E-01 | 28% | 0.09 |
| rs11761290 | 7 | 120932659 | a/g | 0.53 | 1.16 | 2.50E-01 | 28% | 0.09 |
| rs10228676 | 7 | 120932913 | a/g | 0.53 | 1.16 | 2.49E-01 | 28% | 0.09 |
| rs1013711 | 7 | 120943334 | t/c | 0.27 | 0.43 | 6.68E-01 | 24% | 0.10 |
| rs1528351 | 7 | 120955111 | t/c | 0.42 | 0.82 | 4.19E-01 | 23% | 0.11 |
| rs752045 | 8 | 5937538 | a/g | 0.81 | -0.48 | 6.31E-01 | 0% | 0.50 |
| rs2142991 | 10 | 42661111 | t/c | 0.84 | 1.69 | 9.33E-02 | 0% | 0.92 |
| rs2796549 | 10 | 42686043 | a/g | 0.17 | -1.73 | 8.54E-02 | 0% | 0.93 |
| rs3121278 | 10 | 42695652 | t/g | 0.17 | -1.84 | 6.73E-02 | 0% | 0.55 |
| rs7128512 | 11 | 73049947 | a/g | 0.15 | -0.75 | 4.55E-01 | 0% | 0.65 |
| rs6590 | 11 | 73051200 | a/g | 0.10 | -1.40 | 1.64E-01 | 33% | 0.06 |
| rs11235843 | 11 | 73051644 | a/g | 0.10 | -1.40 | 1.65E-01 | 34% | 0.06 |

Adjusted for genomic control (λ)= 1.02
