# Supplementary material for: GWAS analysis of handgrip and lower body strength in older adults in the CHARGE consortium
Source: Aging Cell. 2016 Jun 21;15(5):792–800. doi: 10.1111/acel.12468 (PMC5013019; doi:10.1111/acel.12468)
Supplement: Supplementary file 3 — Appendix S1 Detailed Description of Discovery Cohorts [file ACEL-15-792-s003.docx]

**Supplemental Materials**

**Detailed Description of Discovery Cohorts**

AGES: The AGES–Reykjavik Study is a population-based study of older individuals from the 40-year long Reykjavik Study. Participants were aged between 66 and 96 years and were randomly recruited between 2002 and 2006 from surviving Reykjavik Study members (Tamara B Harris et al. 2007). Informed consent was obtained from all participants. Details of the investigations are described in the study's baseline article.

CHS: The CHS is a population-based cohort study of risk factors for CHD and stroke in adults ≥65 years conducted across four field centers (Fried et al. 1991). The original predominantly Caucasian cohort of 5,201 persons was recruited in 1989-1990 from random samples of the Medicare eligibility lists; subsequently, an additional predominantly African-American cohort of 687 persons was enrolled for a total sample of 5,888. DNA was extracted from blood samples drawn on all participants at their baseline examination in 1989-90. In 2007-2008, genotyping was performed at the General Clinical Research Center's Phenotyping/Genotyping Laboratory at Cedars-Sinai using the Illumina 370CNV BeadChip system on 3980 CHS participants who were free of CVD at baseline, consented to genetic testing, and had DNA available for genotyping

FHS: The Framingham Heart Study was initiated in 1948 to study determinants of cardiovascular disease and other major illnesses. The Original Cohort included 5,209 men and women, aged 28-62 years at enrollment who have undergone routine biennial examinations (DAWBER, MEADORS, and MOORE 1951). In 1971, Offspring of the Original Cohort participants and Offspring spouses including 5,124 men and women, aged 5 to 70 years, were enrolled into the Framingham Offspring Study. Offspring participants have been examined approximately every 4 years. In the 1990s, DNA was obtained for genetic studies from surviving Original Cohort and Offspring participants. All participants provided informed consent for all assessments through the Boston University Medical Center IRB.

Health ABC: Between March 1997 and July 1998, 3,075 black and white men and women aged 70 to 80 were recruited to participate in the Health ABC Study; characteristics of the cohort have been described elsewhere (T B Harris et al. 2000). Medicare beneficiary listings were used to recruit in metropolitan areas surrounding Pittsburgh, Pennsylvania, and Memphis, Tennessee. Eligibility criteria included having no difficulty walking one-quarter of a mile, climbing 10 steps, or performing activities of daily living (transferring, bathing, dressing, and eating); no history of active treatment for cancer in the prior 3 years; and no plans to move from the area within 3 years.

HRS: The Health and Retirement Study (HRS) is a longitudinal survey of a representative sample of Americans over the age of 50.  The current sample is over 26,000 persons in 17,000 households. The study interviews respondents every two years about income and wealth, health and use of health services, work and retirement, and family connections (Sonnega et al. 2014).   DNA was extracted from saliva collected during a face-to-face interview in the respondents' homes.  These data represent respondents who provided DNA samples and signed consent forms in 2006 and 2008.

InCHIANTI: The study participants consisted of men and women, aged 65 and older, who participated in the Invecchiare in Chianti, “Aging in the Chianti Area” (InCHIANTI) study, conducted in two small towns in Tuscany, Italy. The rationale, design, and data collection have been described elsewhere, and the main outcome of this longitudinal study is mobility disability (Ferrucci et al. 2000). Briefly, in August 1998, 1270 people aged 65 years and older were randomly selected from the population registry of Greve in Chianti (pop. 11,709) and Bagno a Ripoli (pop. 4,704), and of 1,256 eligible subjects, 1,155 (90.1%) agreed to participate. Participants received an extensive description of the study and participated after written, informed consent. The study protocol complied with the Declaration of Helsinki and was approved by the Italian National Institute of Research and Care on Aging Ethical Committee and by the Institutional Review Board of the Johns Hopkins University School of Medicine.

LBC: The Lothian Birth Cohort 1921 (LBC1921) cohort consists of 550 relatively healthy individuals, 316 females and 234 males, assessed on cognitive and medical traits at 79 years of age. They were born in 1921, most took part in the Scottish Mental Survey of 1932, and almost all lived independently in the Lothian region (Edinburgh City and surrounding area) in Scotland. When tested, the sample had a mean age of 79.1 years (SD = 0.6). A full description of participant recruitment and testing can be found elsewhere (Deary et al. 2004). Ethics permission for the study was obtained from the Lothian Research Ethics Committee (LREC/1998/4/183). The research was carried out in compliance with the Helsinki Declaration. All subjects gave written, informed consent.

The Lothian Birth Cohort 1936 (LBC1936) consists of 1,091 relatively healthy individuals assessed on cognitive and medical traits at 70 years of age. They were born in 1936, most took part in the Scottish Mental Survey of 1947, and almost all lived independently in the Lothian region of Scotland. The sample of 548 men and 543 women had a mean age 69.6 years (SD = 0.8). A full description of participant recruitment and testing can be found elsewhere (Deary et al. 2007). Ethics permission for the study was obtained from the Multi-Centre Research Ethics Committee for Scotland (MREC/01/0/56) and from Lothian Research Ethics Committee (LBC1936: LREC/2003/2/29). The research was carried out in compliance with the Helsinki Declaration. All subjects gave written, informed consent.

MAP: The Memory and Aging Project (MAP) is a longitudinal clinical-pathologic cohort study of aging. The study enrolls older adults without known dementia who agree to an annual assessment of risk factors, blood donation and a structured clinical evaluation (Bennett et al. 2005). All participants sign an Anatomical Gift Act and agree to the donation of brain, the entire spinal cord and selected nerve and muscles at the time of death. Study participants are primarily recruited from retirement communities throughout northeastern Illinois. Since October 1997, about 1,650 participants have completed their baseline evaluation. The follow-up rate of survivors exceeds 90%, and the autopsy rate exceeds 80%. The Religious Order Study (ROS) is a longitudinal clinical-pathologic cohort study of aging. The study enrolls older adults without known dementia who agree to an annual assessment of risk factors, blood donation and a detailed evaluation (Bennett et al. 2012). All participants sign an Anatomical Gift Act and agree to donation of brain at the time of death. Study participants are primarily Catholic priests, nuns and brothers from about 40 groups in 12 states. Since January 1994, over 1,100 participants completed their baseline evaluation. Both the follow-up rate among survivors and the autopsy rate exceed 90%.

MrOS: The Osteoporotic Fractures in Men Study (MrOS) is a multi-center prospective, longitudinal, observational study of risk factors for vertebral and all non-vertebral fractures in older men, and of the sequelae of fractures in men (Blank et al. 2005; Orwoll et al. 2005). The original specific aims of the study include: (1) to define the skeletal determinants of fracture risk in older men, (2) to define lifestyle and medical factors related to fracture risk, (3) to establish the contribution of fall frequency to fracture risk in older men, (4) to determine to what extent androgen and estrogen concentrations influence fracture risk, (5) to examine the effects of fractures on quality of life, (6) to identify sex differences in the predictors and outcomes of fracture, (7) to collect and store serum, urine and DNA for future analyses as directed by emerging evidence in the fields of aging and skeletal health, and (8) define the extent to which bone mass/fracture risk and prostate diseases are linked. From March 2000 to April 2002, 5994 community dwelling ambulatory men aged 65 years or older were recruited from six communities in the United States (Birmingham, AL; Minneapolis, MN; Palo Alto, CA; Monongahela Valley near Pittsburgh, PA; Portland, OR; and San Diego, CA). Inclusion criteria were designed to provide a study cohort that is representative of the broad population of older men. The MrOS inclusion criteria were: (1) ability to walk without the assistance of another, (2) absence of bilateral hip replacements, (3) ability to provide self-reported data, (4) residence near a clinical site for the duration of the study, (5) absence of a medical condition that (in the judgment of the investigator) would result in imminent death, (6) ability to understand and sign an informed consent, and (7) 65 years or older. To qualify as an enrollee, the participant had to provide written informed consent, complete the self-administered questionnaire (SAQ), attend the clinic visit, and complete at least the anthropometric, DEXA, and vertebral X-ray procedures. The institutional review board at each center approved the study protocol, and written informed consent was obtained from all the participants.

SHIP: The Study of Health in Pomerania (SHIP) is a population based in West Pomerania, the north-east area of Germany (John et al. 2001; Völzke et al. 2011). A sample from the population aged 20 to 79 years was drawn from population registries. First, the three cities of the region (with 17,076 to 65,977 inhabitants) and the 12 towns (with 1,516 to 3,044 inhabitants) were selected, and then 17 out of 97 smaller towns (with less than 1,500 inhabitants), were drawn at random. Second, from each of the selected communities, subjects were drawn at random, proportional to the population size of each community and stratified by age and gender. Only individuals with German citizenship and main residency in the study area were included. Finally, 7,008 subjects were sampled, with 292 persons of each gender in each of the twelve five-year age strata. In order to minimize drop-outs by migration or death, subjects were selected in two waves. The net sample (without migrated or deceased persons) comprised 6,267 eligible subjects. Selected persons received a maximum of three written invitations. In case of non-response, letters were followed by a phone call or by home visits if contact by phone was not possible. The SHIP population finally comprised 4,308 participants (corresponding to a final response of 68.7%). This study includes data of 320 individuals with complete GWAS data who participated in the 10-year follow-up examinations (SHIP-2).

SOF: The Study of Osteoporotic Fractures (SOF) is a prospective multicenter study of risk factors for vertebral and non-vertebral fractures (Cummings et al. 1990). From 1986 to 1987, 9704 community dwelling women aged 65 years or older were recruited from population-based listings in four U.S. areas: Baltimore, Maryland; Minneapolis, Minnesota; Portland, Oregon; and the Monongahela Valley, Pennsylvania. The SOF participants were followed up every four months by postcard or telephone to ascertain the occurrence of falls, fractures and changes in address. The SOF inclusion criteria were: 1) 65 years or older, (2) ability to walk without the assistance of another, (3) absence of bilateral hip replacements, (4) ability to provide self-reported data, (5) residence near a clinical site for the duration of the study, (6) absence of a medical condition that (in the judgment of the investigator) would result in imminent death, and (7) ability to understand and sign an informed consent. To qualify as an enrollee, the participant had to provide written informed consent, complete the self-administered questionnaire (SAQ), attend the clinic visit, and complete at least the anthropometric measures. The institutional review board at each center approved the study protocol, and written informed consent was obtained from all the participants.

TASCOG: TASCOG is a study of cerebrovascular mechanisms underlying gait, balance and cognition in a population-based sample of Tasmanian people aged at least 60 years. Individuals aged 60–86 years (n = 395) living in Southern Tasmania, Australia, were randomly selected from the electoral roll between 2006 and 2008 to participate in the study. Individuals were excluded if they lived in a nursing home, had a contraindication for magnetic resonance scanning (MRI) or were unable to walk without a gait aid18. Participants underwent brain MRI scans and genotyping. DNA was extracted from peripheral blood samples by proteinase K digestion following cell lysis, then phenol-chloroform purification. DNA was genotyped using Illumina Hap370CNV chips at the University of Queensland Diamantina Institute, Princess Alexandra Hospital, Brisbane, Australia, for 370 participants, and call rates were greater than 97% for all samples. Genotypes for 22 individuals were excluded, either because they were closely related to other individuals, they were outliers in a population ancestry analysis or their sex predicted from genotypes did not match sex as recorded in the database. Among the 348 remaining participants with available genome-wide data, after exclusion of 2 participants with dementia, 3 with posterior circulation infarcts on MRI and 3 with insufficient MRI image quality, 340 individuals were available for the present analysis on hippocampal volume.

TUK: The TwinsUK registry now consists of about 12,000 monozygotic (MZ) and dizygotic (DZ) twins aged 18 to 103 years. About 83% of the registry is female (mean age of 55 years). The registry now contains 51% MZ and 49% DZ twins. Between 1992 and 2004, twins were invited for a full comprehensive visit and several project-led studies. More than 7,000 twins responded to some of the annual questionnaires and 5,725 attended a comprehensive visit. Apart from a lifelong lower weight in MZ twins of around 1 kg, all other age-matched characteristics of these volunteer twins were found not to differ from a singleton population-based cohort of British women (Moayyeri et al. 2013). Between April 2004 and May 2007, all the 6,740 active twins on the registry were invited for a 1-day clinical visit, of whom 3,725 twins attended and 1,299 twins posted their blood DNA samples via their general practitioners. The age of participants ranged between 18 and 82 years (mean 52.5 ± 13 years) and 3,299 of the clinic attendants (89%) were female.

**Tissue Sample Description for Expression quantitative trait loci (eQTL) analysis**

Muscle eQTL studies included tissue from Vastus lateralis muscle taken with Bergstrom needles and esophageal and skeletal muscle from the Genotype-Tissue Expression Project GTex (Keildson et al. 2014; *Nature Genetics* 2013). Brain eQTL studies included brain cortex (Heinzen et al. 2008; Webster et al. 2009; Zou et al. 2012), cerebellar cortex (Ramasamy et al. 2014), cerebellum (Gamazon et al. 2013; Gibbs et al. 2010; Kim et al. 2012; Zhang et al. 2013; Zou et al. 2012), frontal cortex (Ramasamy et al. 2014; Gibbs et al. 2010; Kim et al. 2012), gliomas (Shpak et al. 2014), hippocampus (Ramasamy et al. 2014; Kim et al. 2012), inferior olivary nucleus (from medulla) (Ramasamy et al. 2014), intralobular white matter (Ramasamy et al. 2014), occiptal cortex (Ramasamy et al. 2014), parietal lobe (Gamazon et al. 2013), pons (Gibbs et al. 2010), pre-frontal cortex (Kim et al. 2012; Zhang et al. 2013; Colantuoni et al. 2011; Liu et al. 2010), putamen (at the level of anterior commussure) (Ramasamy et al. 2014), substantia nigra (Ramasamy et al. 2014), temporal cortex (Zou et al. 2012; Ramasamy et al. 2014; Gibbs et al. 2010; Kim et al. 2012), thalamus (Kim et al. 2012) and visual cortex (Zhang et al. 2013).

**Funding/Support**

**AGES:** The Age, Gene/Environment Susceptibility Reykjavik Study is funded by NIH contract N01-AG-12100, the NIA Intramural Research Program, Hjartavernd (the Icelandic Heart Association), and the Althingi (the Icelandic Parliament).

**ARIC:** The Atherosclerosis Risk in Communities Study is carried out as a collaborative study supported by National Heart, Lung, and Blood Institute contracts (HHSN268201100005C, HHSN268201100006C, HHSN268201100007C, HHSN268201100008C, HHSN268201100009C, HHSN268201100010C, HHSN268201100011C, and HHSN268201100012C), R01HL087641, R01HL59367 and R01HL086694; National Human Genome Research Institute contract U01HG004402; and National Institutes of Health contract HHSN268200625226C. The authors thank the staff and participants of the ARIC study for their important contributions. Infrastructure was partly supported by Grant Number UL1RR025005, a component of the National Institutes of Health and NIH Roadmap for Medical Research.

**CHS:** This CHS research was supported by NHLBI contracts HHSN268201200036C, HHSN268200800007C, N01HC55222, N01HC85079, N01HC85080, N01HC85081, N01HC85082, N01HC85083, N01HC85086; and NHLBI grants U01HL080295, R01HL087652, R01HL105756, R01HL103612, and R01HL120393 with additional contribution from the National Institute of Neurological Disorders and Stroke (NINDS). Additional support was provided through R01AG023629 from the National Institute on Aging (NIA). A full list of principal CHS investigators and institutions can be found at [CHS-NHLBI.org](http://chs-nhlbi.org/). The provision of genotyping data was supported in part by the National Center for Advancing Translational Sciences, CTSI grant UL1TR000124, and the National Institute of Diabetes and Digestive and Kidney Disease Diabetes Research Center (DRC) grant DK063491 to the Southern California Diabetes Endocrinology Research Center. The content is solely the responsibility of the authors and does not necessarily represent the official views of the National Institutes of Health.

**FHS:** The Framingham Heart Study is supported by the National Institute on Aging (AG08122, AG033193, AG016495). The FHS phenotype-genotype analyses were supported by the National Institute of Aging (R01AG29451). This research was conducted in part using data and resources from the Framingham Heart Study of the National Heart Lung and Blood Institute of the National Institutes of Health and Boston University School of Medicine.   The analyses reflect intellectual input and resource development from the Framingham Heart Study investigators participating in the SNP Health Association Resource (SHARe) project. This work was partially supported by the National Heart, Lung and Blood Institute's Framingham Heart Study (Contract No. N01-HC-25195) and its contract with Affymetrix, Inc for genotyping services (Contract No. N02-HL-6-4278). A portion of this research utilized the Linux Cluster for Genetic Analysis (LinGA-II) funded by the Robert Dawson Evans Endowment of the Department of Medicine at Boston University School of Medicine and Boston Medical Center. Dr. Kiel’s effort was supported by a grant from the National Institute of Arthritis Musculoskeletal and Skin Diseases (R01 AR41398)

**HABC:** The Health ABC Study was supported by NIA contracts N01AG62101, N01AG62103, and N01AG62106 and, in part, by the NIA Intramural Research Program. The genome-wide association study was funded by NIA grant 1R01AG032098-01A1 to Wake Forest University Health Sciences and genotyping services were provided by the Center for Inherited Disease Research (CIDR). CIDR is fully funded through a federal contract from the National Institutes of Health to The Johns Hopkins University, contract number HHSN268200782096C. This study utilized the high-performance computational capabilities of the Biowulf Linux cluster at the National Institutes of Health, Bethesda, Md. (http://biowulf.nih.gov).

**HRS:** HRS is supported by the National Institute on Aging (NIA U01AG009740).  The genotyping was funded as a separate award from the National Institute on Aging (RC2 AG036495).  Our genotyping was conducted by the NIH Center for Inherited Disease Research (CIDR) at Johns Hopkins University.  Genotyping quality control and final preparation of the data were performed by the Genetics Coordinating Center at the University of Washington.

**InCHIANTI:** The InCHIANTI study baseline (1998-2000) was supported as a "targeted project" (ICS110.1/RF97.71) by the Italian Ministry of Health and in part by the U.S. National Institute on Aging (Contracts: 263 MD 9164 and 263 MD 821336).

**LBC:** Phenotype collection in the Lothian Birth Cohort 1921 was supported by the UK Biotechnology and Biological Sciences Research Council (BBSRC), The Royal Society and The Chief Scientist Office of the Scottish Government. Phenotype collection in the Lothian Birth Cohort 1936 was supported by Age UK (The Disconnected Mind project). Genotyping of the cohorts was funded by the BBSRC. The work was undertaken by The University of Edinburgh Centre for Cognitive Ageing and Cognitive Epidemiology, part of the cross council Lifelong Health and Wellbeing Initiative (MR/K026992/1). Funding from the BBSRC and Medical Research Council (MRC) is gratefully acknowledged.

**MAP/ROS:** The Memory and Aging Project is supported by NIA grants R01AG15819, R01AG17917, R01AG24480, R01AG040039 and the Translational Genomics Research Institute. The Religious Orders Study is supported by NIA grants P30AG10161, R01AG15819, and R01AG30146, and the Translational Genomics Research Institute.

**MrOS:** The Osteoporotic Fractures in Men (MrOS) Study is supported by National Institutes of Health funding. The following institutes provide support: the National Institute of Arthritis and Musculoskeletal and Skin Diseases (NIAMS), the National Institute on Aging (NIA), the National Center for Research Resources (NCRR), and NIH Roadmap for Medical Research under the following grant numbers: U01 AR45580, U01 AR45614, U01 AR45632, U01 AR45647, U01 AR45654, U01 AR45583, U01 AG18197, U01-AG027810, and UL1 RR024140. The National Institute of Arthritis and Musculoskeletal and Skin Diseases (NIAMS) provides funding for the MrOS ancillary study ‘Replication of candidate gene associations and bone strength phenotype in MrOS’ under the grant number R01-AR051124. The National Institute of Arthritis and Musculoskeletal and Skin Diseases (NIAMS) provides funding for the MrOS ancillary study

‘GWAS in MrOS and SOF’ under the grant number RC2ARO58973.

**RSI and RSII:** The generation and management of GWAS genotype data for the

Rotterdam Study is supported by the Netherlands Organization of Scientific Research NWO

Investments (nr. 175.010.2005.011, 911-03-012, 050-060-810) and the Research Institute for

Diseases in the Elderly (014-93-015; RIDE2). We thank Pascal Arp, Mila Jhamai, Marijn Verkerk,

Lizbeth Herrera and Marjolein Peters for their help in creating the GWAS database, and Karol Estrada and Maksim V. Struchalin for their support in creation and analysis of imputed data. The Rotterdam Study is funded by Erasmus Medical Center and Erasmus University, Rotterdam, Netherlands Organization for the Health Research and Development (ZonMw), the Research Institute for Diseases in the Elderly (RIDE), the Ministry of Education, Culture and Science, the Ministry for Health, Welfare and Sports, the European Commission (DG XII), and the Municipality of Rotterdam. The authors are grateful to the study participants, the staff from the Rotterdam Study and the participating general practitioners and pharmacists.

**SHIP:** SHIP is part of the Community Medicine Research Network (CMR) of the University of Greifswald, Germany, which is funded by the German Federal Ministry of Education and Research (BMBF; grants 01ZZ9603, 01ZZ0103 and 01ZZ0403), the German Ministry of Cultural Affairs and the Social Ministry of the Federal State of Mecklenburg–West Pomerania, and the network ‘Greifswald Approach to Individualized Medicine (GANI_MED)’ funded by the Federal Ministry of Education and Research (grant 03IS2061A). Genome-wide data have been supported by the Federal Ministry of Education and Research (grant no. 03ZIK012) and a joint grant from Siemens Healthcare, Erlangen, Germany and the Federal State of Mecklenburg- West Pomerania. The University of Greifswald is a member of the Caché Campus program of the InterSystems GmbH.

**SOF:** The Study of Osteoporotic Fractures (SOF) is supported by National Institutes of Health funding. The National Institute on Aging (NIA) provides support under the following grant numbers: R01 AG005407, R01 AR35582, R01 AR35583, R01AR35584, R01 AG005394, R01 AG027574, and R01 AG027576.

**TasCog:** The Tasmanian Study of Gait and Cognition (TASCOG): This study is supported by project grants from the National Health and Medical Research Council of Australia (NHMRC; 403000, 491109 and 606543) and a grant from the Wicking Dementia Education and Research Centre, Hobart. VS is supported by a co-funded NHMRC Career Development Fellowship (1061457) and a Heart Foundation Future Leader Fellowship (ID 100089). MC is supported by an NHRMC Early Career Fellowship (1034483)

**TwinsUK:** The study was funded by the Wellcome Trust; European Community’s Seventh Framework Programme (FP7/2007-2013). The study also receives support from the National Institute for Health Research (NIHR) BioResource Clinical Research Facility and Biomedical Research Centre based at Guy's and St Thomas' NHS Foundation Trust and King's College London. Tim Spector is holder of an ERC Advanced Principal Investigator award. SNP Genotyping was performed by The Wellcome Trust Sanger Institute and National Eye Institute via NIH/CIDR.

**Supplemental References**

Bennett, David A, Julie A Schneider, Zoe Arvanitakis, and Robert S Wilson. 2012. “Overview and Findings from the Religious Orders Study.” *Current Alzheimer Research* 9 (6): 628–45. http://www.pubmedcentral.nih.gov/articlerender.fcgi?artid=3409291&tool=pmcentrez&rendertype=abstract.

Bennett, David A, Julie A Schneider, Aron S Buchman, Carlos Mendes de Leon, Julia L Bienias, and Robert S Wilson. 2005. “The Rush Memory and Aging Project: Study Design and Baseline Characteristics of the Study Cohort.” *Neuroepidemiology* 25 (4): 163–75. doi:10.1159/000087446.

Blank, Janet Babich, Peggy Mannen Cawthon, Mary Lou Carrion-Petersen, Loretta Harper, J Phillip Johnson, Eileen Mitson, and Romelia Ramírez Delay. 2005. “Overview of Recruitment for the Osteoporotic Fractures in Men Study (MrOS).” *Contemporary Clinical Trials* 26 (5): 557–68. doi:10.1016/j.cct.2005.05.005.

Colantuoni, Carlo, Barbara K Lipska, Tianzhang Ye, Thomas M Hyde, Ran Tao, Jeffrey T Leek, Elizabeth A Colantuoni, et al. 2011. “Temporal Dynamics and Genetic Control of Transcription in the Human Prefrontal Cortex.” *Nature* 478 (7370): 519–23. doi:10.1038/nature10524.

Cummings, S R, D M Black, M C Nevitt, W S Browner, J A Cauley, H K Genant, S R Mascioli, J C Scott, D G Seeley, and P Steiger. 1990. “Appendicular Bone Density and Age Predict Hip Fracture in Women. The Study of Osteoporotic Fractures Research Group.” *JAMA* 263 (5): 665–68. http://www.ncbi.nlm.nih.gov/pubmed/2404146.

DAWBER, T R, G F MEADORS, and F E MOORE. 1951. “Epidemiological Approaches to Heart Disease: The Framingham Study.” *American Journal of Public Health and the Nation’s Health* 41 (3): 279–81. http://www.pubmedcentral.nih.gov/articlerender.fcgi?artid=1525365&tool=pmcentrez&rendertype=abstract.

Deary, Ian J, Alan J Gow, Michelle D Taylor, Janie Corley, Caroline Brett, Valerie Wilson, Harry Campbell, et al. 2007. “The Lothian Birth Cohort 1936: A Study to Examine Influences on Cognitive Ageing from Age 11 to Age 70 and Beyond.” *BMC Geriatrics* 7 (January): 28. doi:10.1186/1471-2318-7-28.

Deary, Ian J., Martha C. Whiteman, John M. Starr, Lawrence J. Whalley, and Helen C. Fox. 2004. “The Impact of Childhood Intelligence on Later Life: Following Up the Scottish Mental Surveys of 1932 and 1947.” *Journal of Personality and Social Psychology* 86 (1): 130–47. doi:10.1037/0022-3514.86.1.130.

Ferrucci, L, S Bandinelli, E Benvenuti, A Di Iorio, C Macchi, T B Harris, and J M Guralnik. 2000. “Subsystems Contributing to the Decline in Ability to Walk: Bridging the Gap between Epidemiology and Geriatric Practice in the InCHIANTI Study.” *Journal of the American Geriatrics Society* 48 (12): 1618–25. http://www.ncbi.nlm.nih.gov/pubmed/11129752.

Fried, L P, N O Borhani, P Enright, C D Furberg, J M Gardin, R A Kronmal, L H Kuller, T A Manolio, M B Mittelmark, and A Newman. 1991. “The Cardiovascular Health Study: Design and Rationale.” *Annals of Epidemiology* 1 (3): 263–76. http://www.ncbi.nlm.nih.gov/pubmed/1669507.

Gamazon, E R, J A Badner, L Cheng, C Zhang, D Zhang, N J Cox, E S Gershon, et al. 2013. “Enrichment of Cis-Regulatory Gene Expression SNPs and Methylation Quantitative Trait Loci among Bipolar Disorder Susceptibility Variants.” *Molecular Psychiatry* 18 (3): 340–46. doi:10.1038/mp.2011.174.

Gibbs, J Raphael, Marcel P van der Brug, Dena G Hernandez, Bryan J Traynor, Michael A Nalls, Shiao-Lin Lai, Sampath Arepalli, et al. 2010. “Abundant Quantitative Trait Loci Exist for DNA Methylation and Gene Expression in Human Brain.” *PLoS Genetics* 6 (5): e1000952. doi:10.1371/journal.pgen.1000952.

Harris, T B, M Visser, J Everhart, J Cauley, F Tylavsky, T Fuerst, M Zamboni, et al. 2000. “Waist Circumference and Sagittal Diameter Reflect Total Body Fat Better than Visceral Fat in Older Men and Women. The Health, Aging and Body Composition Study.” *Annals of the New York Academy of Sciences* 904 (May): 462–73. http://www.ncbi.nlm.nih.gov/pubmed/10865790.

Harris, Tamara B, Lenore J Launer, Gudny Eiriksdottir, Olafur Kjartansson, Palmi V Jonsson, Gunnar Sigurdsson, Gudmundur Thorgeirsson, et al. 2007. “Age, Gene/Environment Susceptibility-Reykjavik Study: Multidisciplinary Applied Phenomics.” *American Journal of Epidemiology* 165 (9): 1076–87. doi:10.1093/aje/kwk115.

Heinzen, Erin L, Dongliang Ge, Kenneth D Cronin, Jessica M Maia, Kevin V Shianna, Willow N Gabriel, Kathleen A Welsh-Bohmer, Christine M Hulette, Thomas N Denny, and David B Goldstein. 2008. “Tissue-Specific Genetic Control of Splicing: Implications for the Study of Complex Traits.” *PLoS Biology* 6 (12): e1. doi:10.1371/journal.pbio.1000001.

John, U, B Greiner, E Hensel, J Lüdemann, M Piek, S Sauer, C Adam, et al. 2001. “Study of Health In Pomerania (SHIP): A Health Examination Survey in an East German Region: Objectives and Design.” *Sozial- Und Präventivmedizin* 46 (3): 186–94. http://www.ncbi.nlm.nih.gov/pubmed/11565448.

Keildson, Sarah, Joao Fadista, Claes Ladenvall, Åsa K Hedman, Targ Elgzyri, Kerrin S Small, Elin Grundberg, et al. 2014. “Expression of Phosphofructokinase in Skeletal Muscle Is Influenced by Genetic Variation and Associated with Insulin Sensitivity.” *Diabetes* 63 (3): 1154–65. doi:10.2337/db13-1301.

Kim, S, H Cho, D Lee, and M J Webster. 2012. “Association between SNPs and Gene Expression in Multiple Regions of the Human Brain.” *Translational Psychiatry* 2 (January): e113. doi:10.1038/tp.2012.42.

Liu, C, L Cheng, J A Badner, D Zhang, D W Craig, M Redman, and E S Gershon. 2010. “Whole-Genome Association Mapping of Gene Expression in the Human Prefrontal Cortex.” *Molecular Psychiatry* 15 (8): 779–84. doi:10.1038/mp.2009.128.

Moayyeri, Alireza, Christopher J Hammond, Deborah J Hart, and Timothy D Spector. 2013. “The UK Adult Twin Registry (TwinsUK Resource).” *Twin Research and Human Genetics : The Official Journal of the International Society for Twin Studies* 16 (1): 144–49. doi:10.1017/thg.2012.89.

*Nature Genetics*. 2013. “The Genotype-Tissue Expression (GTEx) Project.” 45 (6): 580–85. doi:10.1038/ng.2653.

Orwoll, Eric, Janet Babich Blank, Elizabeth Barrett-Connor, Jane Cauley, Steven Cummings, Kristine Ensrud, Cora Lewis, et al. 2005. “Design and Baseline Characteristics of the Osteoporotic Fractures in Men (MrOS) Study--a Large Observational Study of the Determinants of Fracture in Older Men.” *Contemporary Clinical Trials* 26 (5): 569–85. doi:10.1016/j.cct.2005.05.006.

Ramasamy, Adaikalavan, Daniah Trabzuni, Sebastian Guelfi, Vibin Varghese, Colin Smith, Robert Walker, Tisham De, et al. 2014. “Genetic Variability in the Regulation of Gene Expression in Ten Regions of the Human Brain.” *Nature Neuroscience* 17 (10): 1418–28. doi:10.1038/nn.3801.

Shpak, Max, Amelia Weber Hall, Marcus M Goldberg, Dakota Z Derryberry, Yunyun Ni, Vishwanath R Iyer, and Matthew C Cowperthwaite. 2014. “An eQTL Analysis of the Human Glioblastoma Multiforme Genome.” *Genomics* 103 (4): 252–63. doi:10.1016/j.ygeno.2014.02.005.

Sonnega, Amanda, Jessica D Faul, Mary Beth Ofstedal, Kenneth M Langa, John W R Phillips, and David R Weir. 2014. “Cohort Profile: The Health and Retirement Study (HRS).” *International Journal of Epidemiology* 43 (2): 576–85. doi:10.1093/ije/dyu067.

Völzke, Henry, Dietrich Alte, Carsten Oliver Schmidt, Dörte Radke, Roberto Lorbeer, Nele Friedrich, Nicole Aumann, et al. 2011. “Cohort Profile: The Study of Health in Pomerania.” *International Journal of Epidemiology* 40 (2): 294–307. doi:10.1093/ije/dyp394.

Webster, Jennifer A, J Raphael Gibbs, Jennifer Clarke, Monika Ray, Weixiong Zhang, Peter Holmans, Kristen Rohrer, et al. 2009. “Genetic Control of Human Brain Transcript Expression in Alzheimer Disease.” *American Journal of Human Genetics* 84 (4): 445–58. doi:10.1016/j.ajhg.2009.03.011.

Zhang, Bin, Chris Gaiteri, Liviu-Gabriel Bodea, Zhi Wang, Joshua McElwee, Alexei A Podtelezhnikov, Chunsheng Zhang, et al. 2013. “Integrated Systems Approach Identifies Genetic Nodes and Networks in Late-Onset Alzheimer’s Disease.” *Cell* 153 (3): 707–20. doi:10.1016/j.cell.2013.03.030.

Zou, Fanggeng, High Seng Chai, Curtis S Younkin, Mariet Allen, Julia Crook, V Shane Pankratz, Minerva M Carrasquillo, et al. 2012. “Brain Expression Genome-Wide Association Study (eGWAS) Identifies Human Disease-Associated Variants.” *PLoS Genetics* 8 (6): e1002707. doi:10.1371/journal.pgen.1002707.
